# Targeting the neuronal calcium sensor DREAM with small-molecules for Huntington’s disease treatment

**DOI:** 10.1038/s41598-019-43677-7

**Published:** 2019-05-13

**Authors:** Alejandro Lopez-Hurtado, Diego A. Peraza, Pilar Cercos, Laura Lagartera, Paz Gonzalez, Xose M. Dopazo, Rosario Herranz, Teresa Gonzalez, Mercedes Martin-Martinez, Britt Mellström, Jose R. Naranjo, Carmen Valenzuela, Marta Gutierrez-Rodriguez

**Affiliations:** 1 Spanish Network for Biomedical Research in Neurodegenerative Diseases (CIBERNED), Instituto de Salud Carlos III, Madrid, Spain; 2 Centro Nacional de Biotecnología, CNB-CSIC, Madrid, Spain; 3 Instituto de Investigaciones Biomedicas Alberto Sols, IIBM, CSIC-UAM, Madrid, Spain; 4 Spanish Network for Biomedical Research in Cardiovascular Research (CIBERCV), Instituto de Salud Carlos III, Madrid, Spain; 5 Instituto de Quimica Medica, IQM-CSIC, Madrid, Spain

**Keywords:** Chemical tools, Medicinal chemistry

## Abstract

DREAM, a neuronal calcium sensor protein, has multiple cellular roles including the regulation of Ca^2+^ and protein homeostasis. We recently showed that reduced DREAM expression or blockade of DREAM activity by repaglinide is neuroprotective in Huntington’s disease (HD). Here we used structure-based drug design to guide the identification of IQM-PC330, which was more potent and had longer lasting effects than repaglinide to inhibit DREAM in cellular and *in vivo* HD models. We disclosed and validated an unexplored ligand binding site, showing Tyr118 and Tyr130 as critical residues for binding and modulation of DREAM activity. IQM-PC330 binding de-repressed c-fos gene expression, silenced the DREAM effect on K_V_4.3 channel gating and blocked the ATF6/DREAM interaction. Our results validate DREAM as a valuable target and propose more effective molecules for HD treatment.

## Introduction

Huntington’s disease (HD) is currently an incurable, progressive neurodegenerative disorder caused by the expansion of CAG triplets in the huntingtin (HTT) gene^1^. Disease symptoms include involuntary and repetitive choreic movements, psychological dysfunction and cognitive impairment, related to a progressive functional loss and degeneration of striatal and cortico-striatal projecting neurons^2^. Although the genetic causes are well-defined, the disease mechanism mediating late onset and progression are poorly understood. Several interconnected pathways that involve altered protein degradation and Ca^2+^ homeostasis have been proposed^3^.

**Figure 1:**
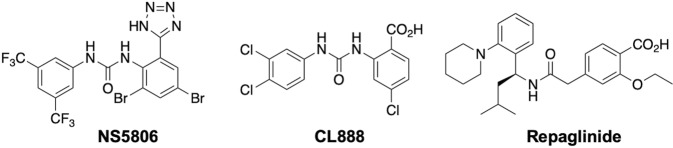
Structure of the previously reported DREAM ligands.

The downstream regulatory element antagonist modulator (DREAM)^4^ belongs to the neuronal calcium sensor family. This protein is also known as KChIP3 or calsenilin, because of its interaction with K_V_4 potassium channels^5^ or presenilins^6^, respectively. Through protein-DNA and protein-protein interactions, DREAM is a key regulator of many cellular functions including Ca^2+^ and protein homeostasis^7,8^. Ca^2+^, arachidonic acid and small molecules that bind to DREAM regulate these protein-protein interactions and thus regulate DREAM function^9^. To date, three molecules have been shown to bind and modulate DREAM: NS5806^10^, repaglinide^11^ and CL-888^11^ (Fig. 1).

We recently found that expression of DREAM is reduced in HD mouse models and in HD patients^11^. DREAM down regulation was observed shortly after birth in the mouse models, and led to endogenous neuroprotection^11^. Chronic administration of repaglinide, a drug commonly used to stimulate insulin secretion, delayed onset of motor dysfunction and cognitive impairment and prolonged life span in mouse HD models, though it was less efficient in advanced disease stages^11,12^. The mechanism involves the interaction between DREAM and the unfolded protein response (UPR) sensor activating transcription factor 6 (ATF6)^11^. These findings suggested a promising approach for therapeutic intervention in HD and showed the need for new more potent DREAM ligands with longer-lasting effects.

The aim of this work was to identify novel DREAM ligands as useful chemical tools to get insights into DREAM biological roles and for the future development of lead compounds for new Huntington’s disease treatments. Using a multidisciplinary approach that involved structure-based drug design, synthesis, surface plasmon resonance, single-site directed mutagenesis, whole-cell patch-clamp electrophysiology, survival after oxidative stress in sensitized STHdhQ^111/111^ cells, co-immunoprecipitation experiments and *in vivo* assays in R6/1 mice, a Huntington’s disease mouse model, we have identified a promising new DREAM ligand, IQM-PC330, which was more potent and had longer lasting effects than the previously reported DREAM ligands.

## Results

### Identification of novel DREAM ligands through structure-based design

Using the CL-888 molecule as starting point, we applied structure-based chemical design strategies to generate a small library of DREAM ligands. We replaced the urea linker in CL-888 with an amide linker; the resulting compound, IQM-PC205 (**2**), showed improved affinity for DREAM in surface plasmon resonance (SPR) assays. We then undertook molecular docking studies to identify the determinants of IQM-PC205 binding. In the absence of experimental three-dimensional structure of the human DREAM protein (hDREAM), we built homology models of DREAM C-terminal region based on the NMR structure of mouse DREAM^13^ (pdb code 2JUL, 15 structures, mDREAM) and the X-ray structure of the KChIP1-K_V_4.3 N-terminal domain complex^14^ (pdb code 2I2R). The sequence identity of mDREAM and KChIP1 with the hDREAM C-terminal region is 93% and 69%, respectively. Comparison of the structure of 2I2R^14^ with those of isolated KChIP1^15^ or mDREAM^13^ (2JUL) indicated a reordering of KChIP1 in 2I2R that generates a site to accommodate the K_V_4.3 N-terminal helix. Five homology hDREAM models were built using the Schrödinger’s Prime module, four based on 2JUL and one on 2I2R. These models provided several protein conformations for the docking studies.

**Figure 2:**
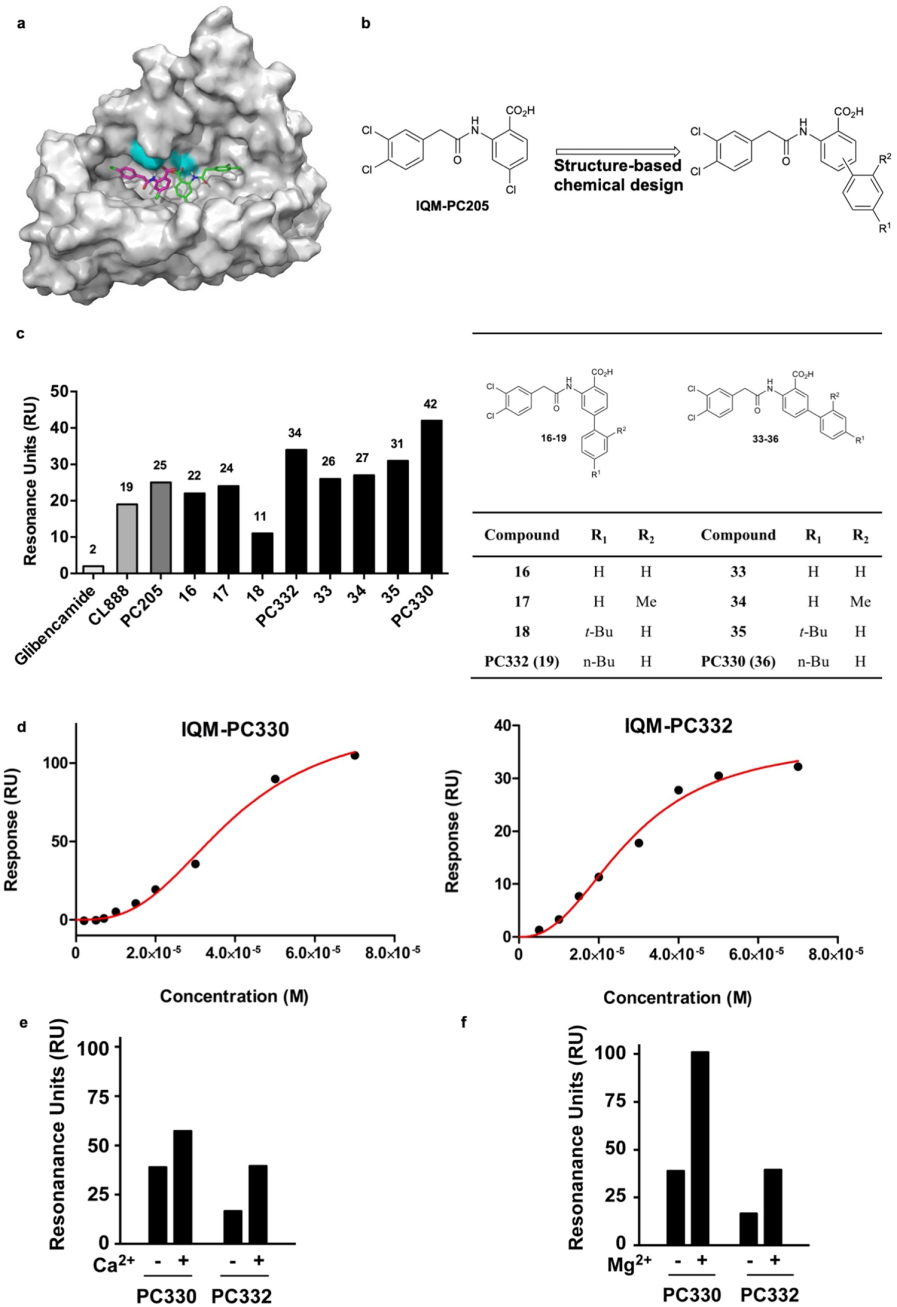
Novel DREAM ligands by structure-based chemical design. (**a**) Superposition of two different binding poses of IQM-PC205 with DREAM. Locations of the Tyr118 and Tyr130 residues are indicated (cyan). (**b)** Design approach and chemical structures. (**c**) Direct binding of selected compounds (**16–18**, IQM-PC332, **33–35**, IQM-PC330) to immobilized DREAM in comparison with the hit IQM-PC205, CL-888 (positive control) and glibenclamide (Gb, negative control) determined by SPR spectroscopy. RU, resonance units. (**d**) SPR binding plot. IQM-PC330 K_D_ = 3.9 ± 0.4 · 10^−5^ M; IQM-PC332 K_D_ = 2.8 ± 0.3 · 10^−5^ M. (**e**) Calcium concentration dependency of the IQM-PC compounds to bind DREAM (**f**) Magnesium concentration dependency of the IQM-PC compounds to bind DREAM.

As there is no experimental information regarding binding pockets for DREAM inhibitors, we used SiteMap (Schrödinger Suite) to aid in predicting potential sites suitable for small molecules. Based on the ranking of these sites and visual inspection, we selected a cavity around Tyr118 and Tyr130, the residues approximately at the center of a large hydrophobic cleft in hDREAM models. Compound IQM-PC205 was docked into this site using Glide and the induced fit docking protocol (IFD). Docking studies indicated multiple binding sites for IQM-PC205 (Fig. 2a), probably due to the large size of the hydrophobic cleft that allows a small molecule such as IQM-PC205 to adopt different binding modes. Tyr118 and/or Tyr130 were nonetheless implicated in most of these theoretical binding sites. Inspection of the cleft topology suggested that the introduction of an additional phenyl ring in IQM-PC205 could provide the necessary features for an efficient interaction with DREAM. Based on this analysis, we designed a small virtual compound library (Fig. 2b). IFD studies of this library and hDREAM models provided more reliable binding poses, and suggested that the introduction of substituents like Me, *t*-Bu or *n*-Bu at R^1^ could further improve binding. Eight potential hits (**16–18**, IQM-PC332 (**19**), **33–35**, IQM-PC330 (**36**)) were selected for synthesis.

**Figure 3:**
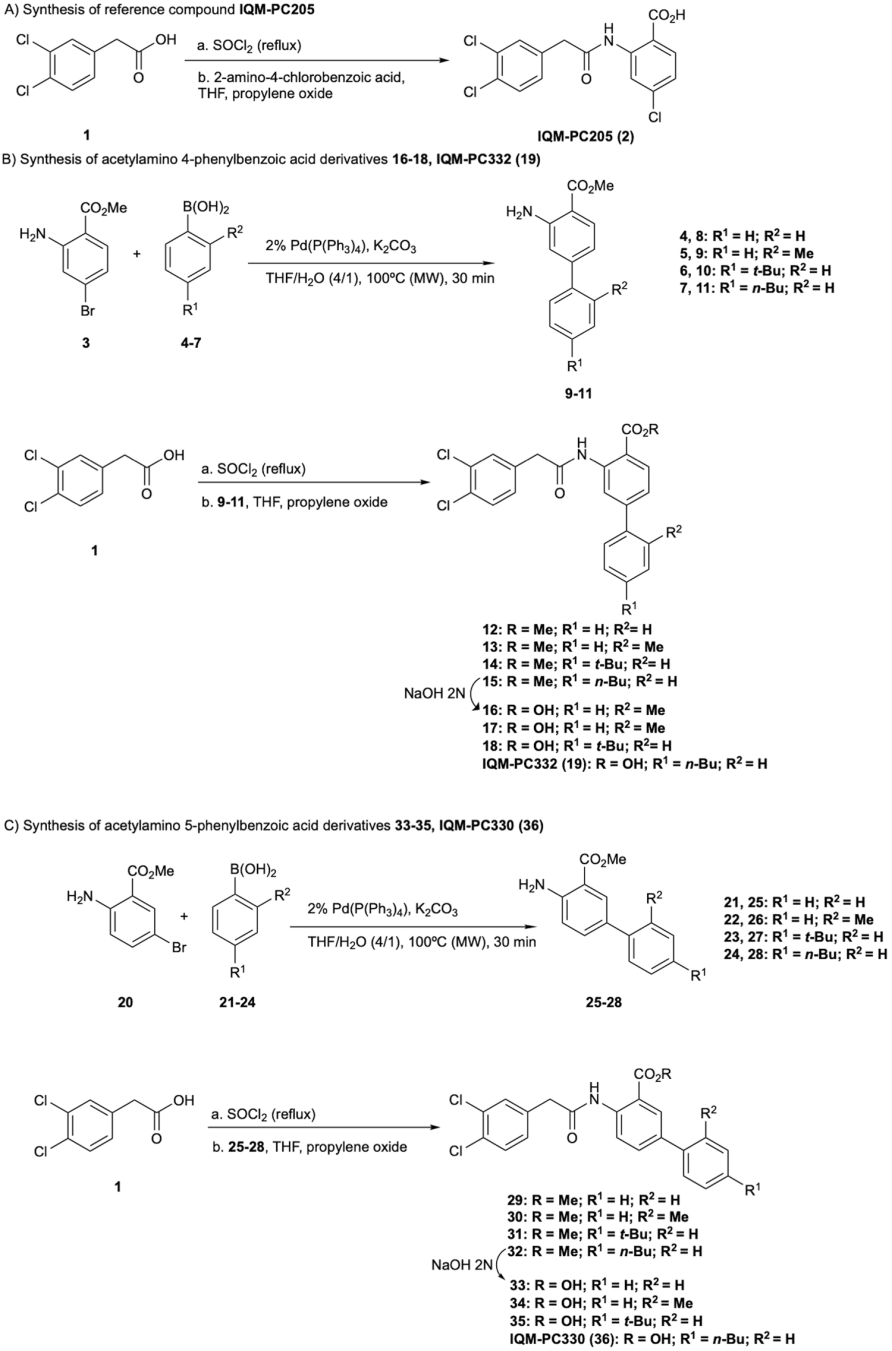
Synthesis of the reference compound **2** and the selected compounds **16–18**, **IQM-PC332**, **33–35** and **IQM-PC330**.

The selected compounds **16–19** and **33–36** were prepared in three steps starting from the commercially available 2-(3,4-dichlorophenyl)acetic acid **1** and the biphenyl intermediates **8–11** and **25–28** (Fig. 3). The biphenyl intermediates **8–11** and **25–28** were obtained by a Suzuki cross-coupling reaction between the boronic acid, **4–7** or **21–24**, and the corresponding bromobenzoic acid derivative (**3** or **20**). The amide formation between the acid chloride, generated *in situ* from **1** by reaction with SOCl_2_, and the biphenyl intermediates led the methyl ester derivatives **9–11** and **25–28**. The ester hydrolysis gave the final compounds **16–19** and **33–36** with good yields and high purities. Surface plasmon resonance assays confirmed the binding of these compounds and identified IQM-PC330 (**36**, K_D_ = 3.9 ± 0.4 · 10^−5^ M) and IQM-PC332 (**19**, K_D_ = 2.8 ± 0.3 · 10^−5^ M), which bear an *n*-Bu moiety at R^2^, as the most promising DREAM-ligands for subsequent biological evaluation (Fig. 2c,d and Supplementary Fig. 1). Furthermore, both compounds bound to DREAM in a calcium and magnesium concentration-dependent manner (Fig. 2e,f).

### Identification of the binding site

**Figure 4:**
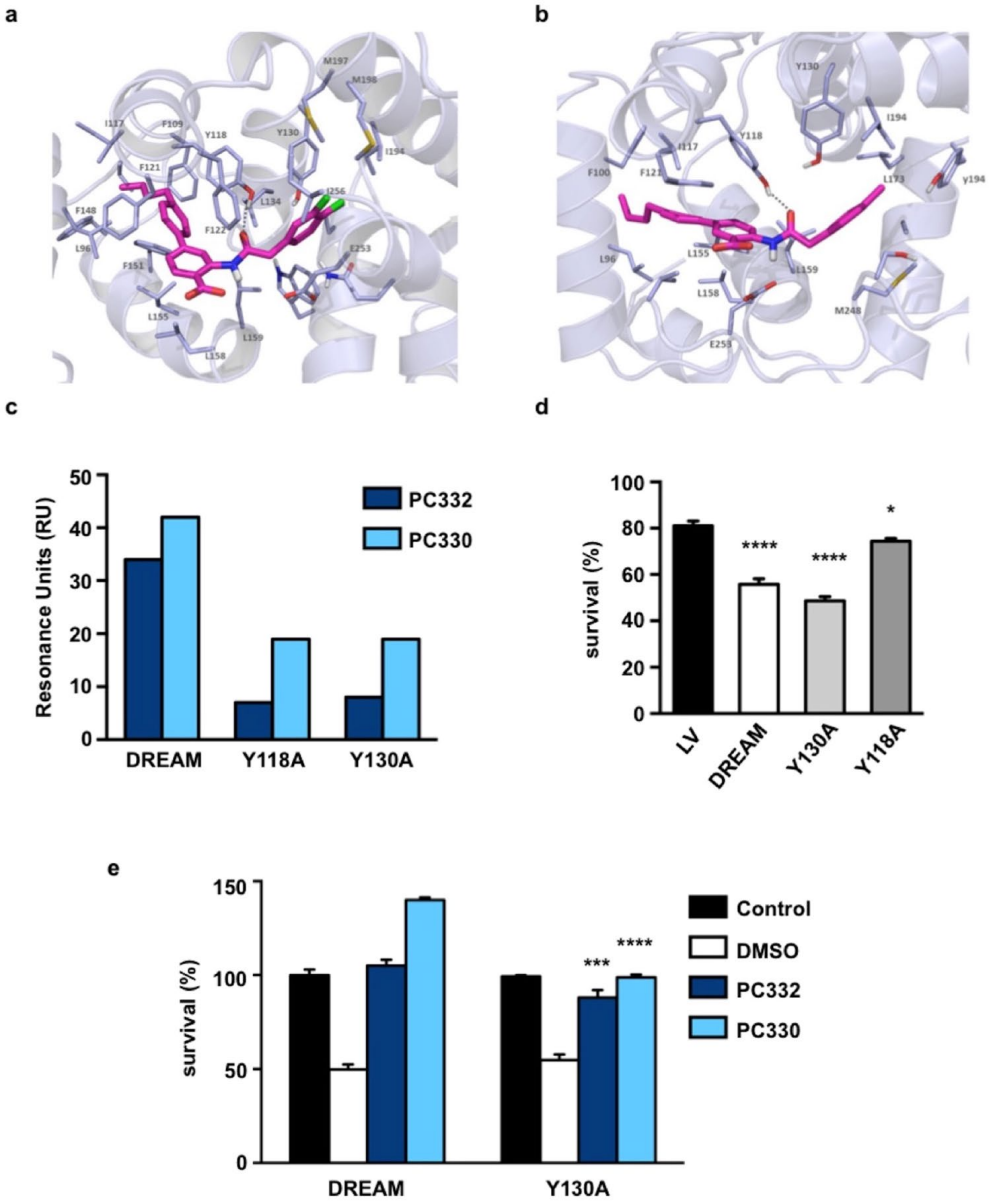
Identification of the binding site. Molecular docking model of IQM-PC332 (**a**) and IQM-PC330 (**b**) in complex with DREAM. Amino acids within 4 Å of the ligand are shown; yellow dashed lines indicated hydrogen bonds. For clarity non-polar hydrogens are not shown. (**c**) Direct binding of IQM-PC332 and IQM-PC330 in SPR assays to immobilized wtDREAM compared to Tyr118Ala and Tyr130Ala DREAM mutants. (**d**) Comparative response of sensitized STHdhQ^111/111^ cells to H_2_O_2_-induced oxidative stress after overexpression of wild type, Tyr118Ala or Tyr130Ala DREAM. One-way ANOVA with Holm-Sidak’s multiple comparison test (n = 6). ****p < 0.0001, *p < 0.05 vs empty vector (LV). (**e**) Effect of vehicle (DMSO), 130 nM IQM-PC332 or 40 nM IQM-PC330 in wild type DREAM- (DREAM) or Tyr130Ala DREAM-sensitized STHdhQ^111/111^ cells (Y130A). Control bar represents DREAM- or Tyr130Ala DREAM sensitized STHdhQ^111/111^ cells non-exposed to H_2_O_2_, respectively. The concentrations of IQM-PC compounds used in these experiments correspond to the minimum concentration able to fully reverse the effect of H_2_O_2_ exposure. Non-parametric Mann-Whitney t-test *p < 0.05 vs corresponding compound in wild type DREAM sensitized STHdhQ^111/111^ cells.

To analyze the binding mode of the new DREAM ligands, we selected IQM-PC330 and IQM-PC332 for simulated annealing molecular dynamics (MD) studies. Based on the IFD analysis, the best binding poses were selected as starting structures for the MD simulations using Amber16 and the FF12SB force field. The DREAM ligand complexes were solvated in an octahedral box using TIP3P water molecules. The MD studies revealed that compounds IQM-PC330 and IQM-PC332 were accommodated within the hydrophobic cleft, mainly through hydrophobic interactions (Fig. 4a,b). In addition, a hydrogen bond was observed between the hydroxyl group of Tyr118 and the IQM-PC330 and IQM-PC332 amide carbonyl group, and another hydrogen bond between the carboxylic moiety of IQM-PC332 and the NH_2_ group of Arg160 side chain. IFD studies suggested a hydrogen bond with Tyr130. This interaction, however, was not confirmed by MD simulation. Instead, Tyr130 interacts with Tyr118 and places this residue in the right position to form the hydrogen bond with IQM-PC ligands. A detailed analysis of the MD studies indicated that the *n*-butyl-biphenyl group of IQM-PC330 and IQM-PC332 is tightly packed, establishing hydrophobic contacts with Leu96, Phe100, Ile117, Tyr118, Phe121, Phe151, Leu155, Leu159, and the hydrophobic part of Glu103. The largest variation is observed in the interactions of the di-chloro-phenyl group, which also showed a greater mobility in the MD simulation. Nevertheless, both ligands share some of the interacting residues as Tyr130 and Leu158. Besides, IQM-PC330 establishes additional interactions with Trp169, Ala170 and Met249, while IQM-PC332 is closed to Leu159, Ile194 and Ile256.

Based on these MD predictions, residues Tyr118 and Tyr130 were selected for mutagenesis studies, and single-site Tyr-to-Ala mutants were prepared. SPR assays using single-site DREAM mutants Tyr118Ala or Tyr130Ala showed a substantial decrease in the binding of the two ligands compared to wild type DREAM (Fig. 4c).

To verify the importance of Tyr118 and Tyr130 residues in DREAM for binding of IQM-PC332 and IQM-PC330, in a biological context, we used DREAM-sensitized STHdhQ^111/111^ neuroblastoma cells. Previously, we have shown that restoring DREAM levels in STHdhQ^111/111^ cells sensitized these cells to oxidative stress and that repaglinide is able to reverse this sensitization and improves survival after hydrogen peroxide exposure^11^. Overexpression of wild type DREAM or any of the Tyr-to-Ala mutants resulted in similar levels of overexpressed protein (Supplementary Fig. 2), nonetheless the DREAM-Tyr118Ala mutant sensitized STHdhQ^111/111^ cells to a much lesser extent, which suggested that this mutation compromises the biological activity of DREAM in this assay (Fig. 4d). Thus, we compared the effect of the compounds in DREAM Tyr130Ala-sensitized STHdhQ^111/111^ and wild type DREAM-sensitized STHdhQ^111/111^ cells. In support of SPR assay data, reduced IQM-PC330 or IQM-PC332 binding to DREAM Tyr130Ala led these compounds to be less effective in protecting DREAM Tyr130Ala-sensitized STHdhQ^111/111^ cells after oxidative stress damage by hydrogen peroxide (Fig. 4e). Taken together, these data support the importance of Tyr118 and Tyr130 for the ligand binding to DREAM, whereas the function of the DREAM protein is compromised in the absence of Tyr118.

### IQM-PC compounds interfere with DREAM repressor function without affecting DREAM oligomerization

**Figure 5:**
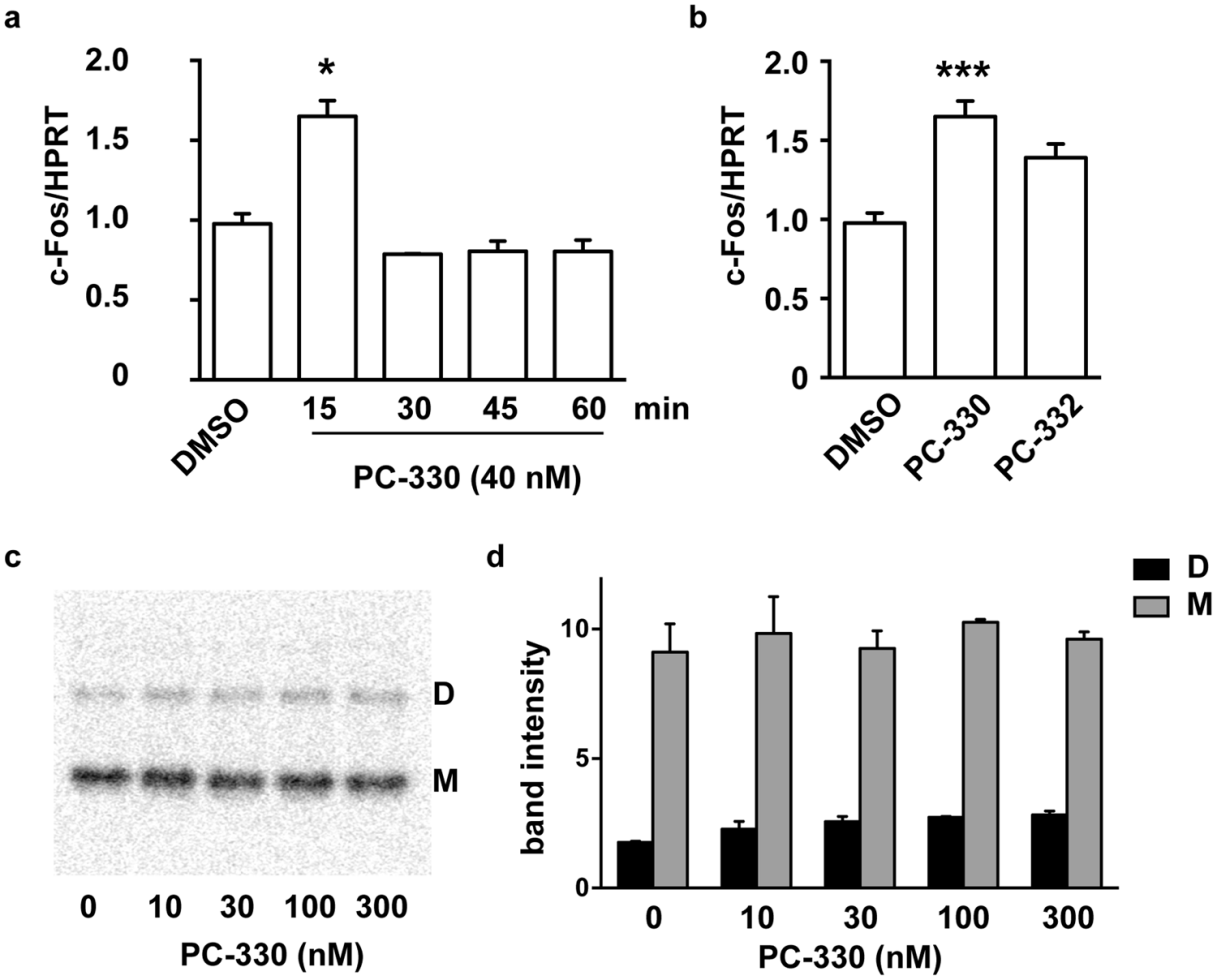
IQM-PC compounds activate c-fos expression without affecting DREAM oligomerization *in vitro*. Quantitative RT-PCR analysis of c-fos mRNA levels in STHdhQ^7/7^ neuroblastoma cells (**a**) at different times after the exposure to IQM-PC330 (40 nM) and (**b**) 15 minutes after addition of IQM-PC330 (40 nM) or IQM-PC332 (130 nM). *p < 0.05 and ***p < 0.001, vs DMSO treated cells. (Non-parametric One-way ANOVA, Dunn’s multiple comparison post-test, n = 2–11). (**c**) Blue native polyacrylamide gel electrophoresis of recombinant DREAM (amino acids 71–256) incubated during 10 min with increasing concentrations of IQM-PC330 before electrophoresis. Bands corresponding to dimer (D) and monomer (M) DREAM are indicated. (**d**) Quantification of band intensity (duplicates, mean ± SEM).

Since binding of IQM-PC compounds to DREAM is Ca^2+^-dependent, we could not directly assess their effect on DREAM binding to DNA using standard *in vitro* methods like the band-shift assay. Instead, to answer the question of a potential effect of IQM-PC330 on DREAM-mediated transcription we first investigated potential changes in basal DREAM-mediated transcription induced by IQM-PC compounds. Previous results from our group have shown that in basal conditions DREAM controls the expression of several immediate-early genes including c-fos^4,16^. Exposure to IQM-PC330 induced a rapid and transient increase of c-fos mRNA levels in STHdhQ^7/7^ neuroblastoma cells (Fig. 5a). The effect peaked at 15 min after IQM-PC330 exposure and was not observed 30 min after treatment (Fig. 5a). Both IQM-PC330 and -332 induced similar effects though IQM-PC330 was noticeably more potent (Fig. 5b). These data suggest that IQM-PC compounds could alter DREAM transcriptional repressor activity. Whether this effect involves an action at the DNA binding site can not be confirmed or ruled out at present.

DREAM binds to specific sites in the DNA, the DRE site, as a tetramer and represses transcription of target genes in basal, non-stimulated conditions^4^. Upon stimulation of the cell and calcium entry in the nucleus, DREAM unbinds from DNA, a process that involves the transition to DREAM dimers and monomers^13^. As DREAM oligomerization is required to form the DNA-bound DREAM tetramer, we hypothesized that binding of IQM-PC compounds could affect DREAM oligomerization and so modify DREAM-mediated transcriptional repression. To test this mechanism, we used recombinant DREAM protein (rDREAM) and blue native (BN) (non-denaturing and non-reducing) polyacrylamide gels. In the presence of Ca^2+^, rDREAM migrates in blue native gels as dimer and monomer (Fig. 5c). Incubation of rDREAM with IQM-PC330 did not significantly modify the ratio dimer/monomer in these *in vitro* conditions (Fig. 5d). Whether IQM-PC330 could affect DREAM oligomerization *in vivo* to de-repress basal immediate early gene expression or whether other mechanism(s) is/are responsible of this effect is presently unknown.

### IQM-PC compounds modulate the gating of K_V_4.3/DREAM channels

**Figure 6:**
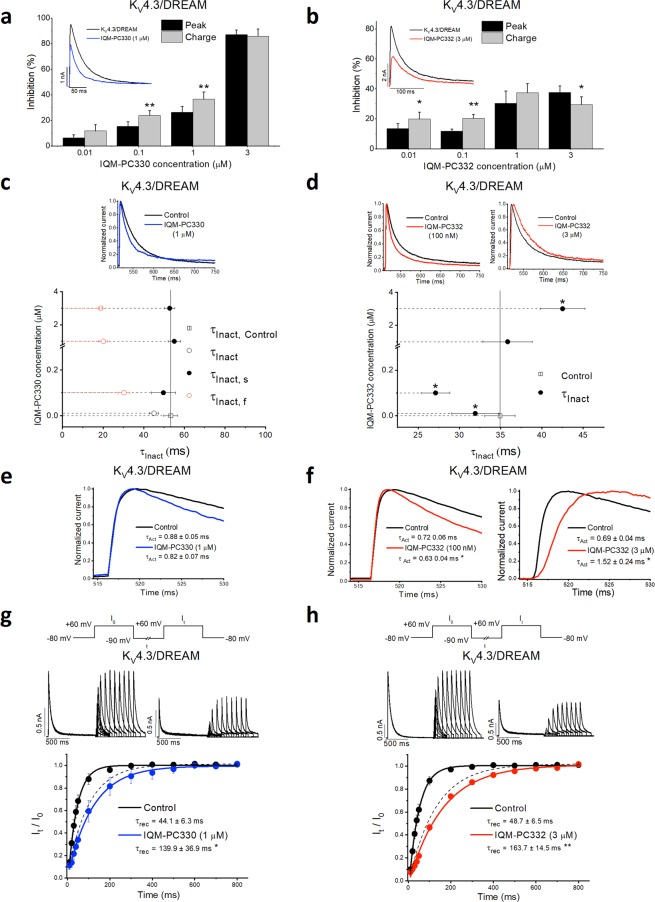
Electrophysiological effects of IQM-PC330 and IQM-PC332 on K_V_4.3 and K_V_4.3/DREAM channels. (**a,b**) Bar charts comparing the inhibition of K_V_4.3/DREAM currents measured at the maximum peak current and at the charge produced by IQM-PC330 and IQM-PC332. Insets show original current recordings. (**c,d**) Graphics showing the changes in the time constant of inactivation (τ_Inact_) at different concentrations of IQM-PC330 and IQM-PC332. IQM-PC330 accelerates the τ_Inact_ making the monoexponential process observed under control conditions in a biexponential one with a fast and a slow τ_Inact_ (τ_Inact,f_ and τ_Inact,s_, respectively). In both graphs, the solid line represents the mean control value of the τ_Inact_. Insets show original normalized current records: control (black line) and with IQM-PC330 (blue line) or IQM-PC332 (red line). Note that IQM-PC332 (100 nM) accelerates the inactivation and at 3 μM slows down this process). (**e,f**) Effects of IQM-PC330 and IQM-PC332 (100 nM and 3 μM) on the activation kinetics. Note that IQM-PC332 (100 nM) accelerates the activation and at 3 μM slows down this process. (**g,h**) Effects of IQM-PC330 and IQM-PC332 on the recovery process. Dashed lines represent the recovery process of K_V_4.3 current without DREAM. Note that both compounds slow the recovery process, reverting DREAM effects.

DREAM is a regulatory subunit of K_V_4.3 channels, which increases the traffic of these channels to the membrane. Also, DREAM modifies the gating of K_V_4.3 channels, delaying their inactivation kinetics, and accelerating their activation and recovery kinetics from inactivation^5,11^. Binding of repaglinide or CL-888 to DREAM modifies the gating properties of the K_V_4.3/DREAM channel complex^11^. We, therefore, analyzed the effects of IQM-PC330 and IQM-PC332 on the gating of the K_V_4.3/DREAM channel complex. Both compounds inhibited the K_V_4.3/DREAM current with IC_50_ values of 1.6 μM (n = 31) and 6.8 μM (n = 45), respectively, measured as the inhibition of the amount of charge crossing the membrane (Fig. 6a,b; Supplementary Fig. 3a,b). Inhibition was also measured as the decrease in the maximum current amplitude (peak current). The inhibitory effect of IQM-PC330 on the K_V_4.3/DREAM channels was greater in the amount of charge crossing the membrane than in the peak amplitude (Fig. 6a,b), probably due to the acceleration of the inactivation kinetics of the current induced by this compound (Fig. 6c, Supplementary Table 1); indeed, IQM-PC330 converted the monoexponential inactivation process of K_V_4.3/DREAM current to biexponential. Inhibition induced by IQM-PC332 at the lowest concentrations tested was also greater when measured as the reduction in the charge; however, at concentrations >1 μM, the inhibition was greater when measured at the maximum peak current (Fig. 6b). These effects were probably due to the biphasic effects of IQM-PC332 on the inactivation kinetics. In fact, low IQM-PC332 concentrations (0.01 to 0.1 μM) accelerated the inactivation kinetics, whereas higher concentrations slowed it down (Fig. 6d, Supplementary Table 1). Since DREAM slows K_V_4.3 inactivation, and these compounds modified the kinetics of K_V_4.3/DREAM current but not the K_V_4.3 current alone (Supplementary Fig. 4a–d and Supplementary Table 1), these results suggest that they bind to DREAM and reverse its effects on the channels.

IQM-PC330 did not modify the activation kinetics of K_V_4.3/DREAM current (Fig. 6e), whereas IQM-PC332 had a dual effect. At concentrations <1 μM, IQM-PC332 accelerated the activation kinetics of K_V_4.3/DREAM current, whereas at concentrations >1 μM slowed it (Fig. 6f). DREAM accelerates activation of K_V_4.3 channels, and the effect of IQM-PC332 appeared to reverse the DREAM effect on channel gating. Neither IQM-PC330 nor IQM-PC332 modified the activation kinetics of K_V_4.3 current without DREAM (Supplementary Fig. 4a–d), again suggesting that their effects on the activation kinetics of these channels are due to interaction with DREAM. IQM-PC332 interaction with DREAM is likely produced through the closed state of the K_V_4.3/DREAM complex, since the compound delays the activation and inactivation processes, and transition to the inactivated state of K_V_4.3 channels is produced mainly through the closed state^17^.

One of the most important DREAM-induced effects on the K_V_4.3 current is the acceleration of the recovery kinetics from inactivation (from 92.7 ± 10.7 ms to 44.4 ± 3.8 ms alone and with DREAM, respectively; n = 25, *p* < 0.01). Both, IQM-PC330 and IQM-PC332 slowed this process significantly when cells were transfected with K_V_4.3/DREAM (Fig. 6g,h). Both compounds slightly slowed the recovery from inactivation process of K_V_4.3 channels inactivation, albeit not significantly (IQM-PC330, 112.8 ± 24.8 vs. 238.3 ± 63.2 ms, *n* = 7, *p* > 0.05; IQM-PC332, 133.8 ± 15.5 vs. 240.8 ± 62.6 ms, *n* = 7, *p* > 0.05) (Supplementary Fig. 4e,f). Recovery from inactivation of K_V_4.3/DREAM channels in the presence of IQM-PC330 and IQM-PC332 was comparable to that observed for K_V_4.3 channels without DREAM (Fig. 6g,h, dashed lines). These results thus suggest that both compounds act by preventing the effects of DREAM on K_V_4.3 channels.

Finally, we studied the effect of the Tyr to Ala mutations in K_V_4.3/DREAM channel complex. Expression of DREAM Tyr118Ala and DREAM Tyr130Ala increased the amplitude of K_V_4.3 current similarly to DREAM wt. Both DREAM mutants accelerated the kinetics of the recovery from inactivation of K_V_4.3 channels in the absence of DREAM, although to a lesser extent than DREAM wt (Table 1). The activation curve of K_V_4.3/DREAM Tyr118Ala channels was shifted to more negative membrane potentials, without changes in the slope. This mutant did not modify the voltage dependent inactivation curve. On the contrary, the activation curve of K_V_4.3/DREAM Tyr130Ala channels was not modified, whereas the inactivation curve was shifted to more positive potentials, without changes in the slope. Both DREAM mutants slowed the kinetics of activation, inactivation and recovery from inactivation, when compared to K_V_4.3/DREAM wt channels (Table 1).

**Table 1:** Electrophysiological characteristics of K_V_4.3/DREAM wt, K_V_4.3/DREAM Y118A and K_V_4.3/DREAM Y130A.

DREAM	E_h,Act_ (mV)	*s*_Act_ (mV)	E_h,Inact_ (mV)	*s*_*Inact*_ (mV)	τ_Act_ (ms)	τ_Inact_ (ms)	τ_Rec_ (ms)
wt	1.1 ± 1.6	16.3 ± 0.2	−34.6 ± 1.7	4.6 ± 0.2	0.66 ± 0.05	45.1 ± 3.7	44.4 ± 3.8
Y118A	−4.3 ± 1.3***	16.7 ± 0.3	−36.3 ± 1.7	4.8 ± 0.1	0.80 ± 0.04*	90.4 ± 4.1***	57.9 ± 4.7*
Y130A	2.2 ± 1.3	16.7 ± 0.3	−26.7 ± 1.1**	4.8 ± 0.1	0.87 ± 0.05*	63.3 ± 3.7***	61.6 ± 5.3**

E_h,Act_: Midpoint of the activation curve, *s*_Act_: Slope of the activation curve, E_h,Inact_: Midpoint of the inactivation curve, *s*_Inact_: Slope of the inactivation curve, τ_Act_: Activation time constant, τ_Inact_: Inactivation time constant, τ_Rec_: Time constant of recovery from inactivation.

*p < 0.05 vs K_V_4.3/DREAM wt, **p < 0.01 vs K_V_4.3/DREAM wt, ***p < 0.001 vs K_V_4.3/DREAM wt of n = 18, 30 (DREAM Y118A) and 23 (DREAM Y130A) experiments.

**Figure 7:**
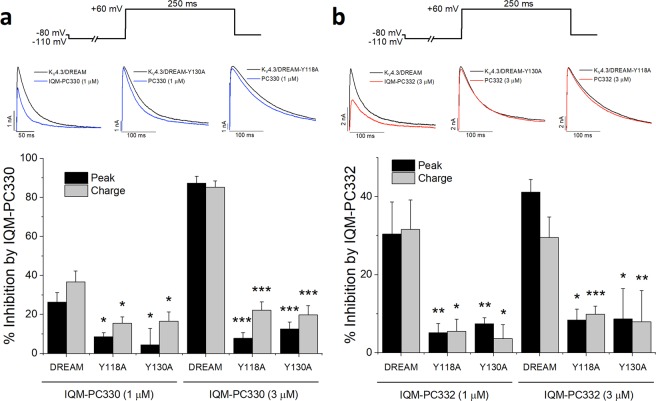
Electrophysiological effects of IQM-PC330 and IQM-PC332 on K_V_4.3/DREAM wt and K_V_4.3/DREAM mutant channels. Top panels depict original records obtained with or without IQM-PC330 (**a**) or IQM-PC332 (**b**) in K_V_4.3/DREAM wt and in K_V_4.3/DREAM Y118A or K_V_4.3/DREAM Y130A channels. Block produced by IQM-PC330 (1 and 3 μM) and IQM-PC332 (1 and 3 μM) of K_V_4.3/DREAM Y118A and K_V_4.3/DREAM Y130A channels measured at the peak and at the charge. Data are shown as mean ± SEM. *p < 0.05 vs block produced in K_V_4.3/DREAM wt channels; **p < 0.01 vs block produced in K_V_4.3/DREAM wt channels; ***p < 0.001 vs block produced in K_V_4.3/DREAM wt channels.

In K_V_4.3/DREAM-Tyr118Ala and K_V_4.3/DREAM Tyr130Ala channels, two concentrations (1 and 3 μM) of each IQM-PC compound were tested (Fig. 7). As it can be observed in Fig. 7, both concentrations produced a negligible inhibition of the current measured both at the charge or at the peak current. As expected from the slight block produced by IQM-PC330 or IQM-PC332, they did not modify the activation and inactivation kinetics in contrast to that recorded in K_V_4.3/DREAM wt channels (Fig. 7a,b). These results are in agreement with those from MD, SPR and in sensitized STHdhQ^111/111^ cells and point out the importance of these two Tyr in the binding site of IQM-PC compounds to DREAM.

### IQM-PC330 promotes ATF6-dependent transcriptional activation

We previously reported that chronic administration of repaglinide delays the appearance of motor symptoms in R6/2 mice^11^. The mechanism involves the displacement of the DREAM-ATF6 interaction, the increase in ATF6 processing and transcriptional activity and the potentiation of the pro-survival phase of the UPR^11^. Here, we analyzed the effect of IQM-PC compounds on the DREAM-ATF6 interaction to test whether these compounds also could have a protective effect for R6/1 symptoms. To limit the number of R6/1 mice necessary for these experiments, we chose IQM-PC330 since *in vitro* and *in cellula* assays supported this compound as a better candidate for *in vivo* assays.

**Figure 8:**
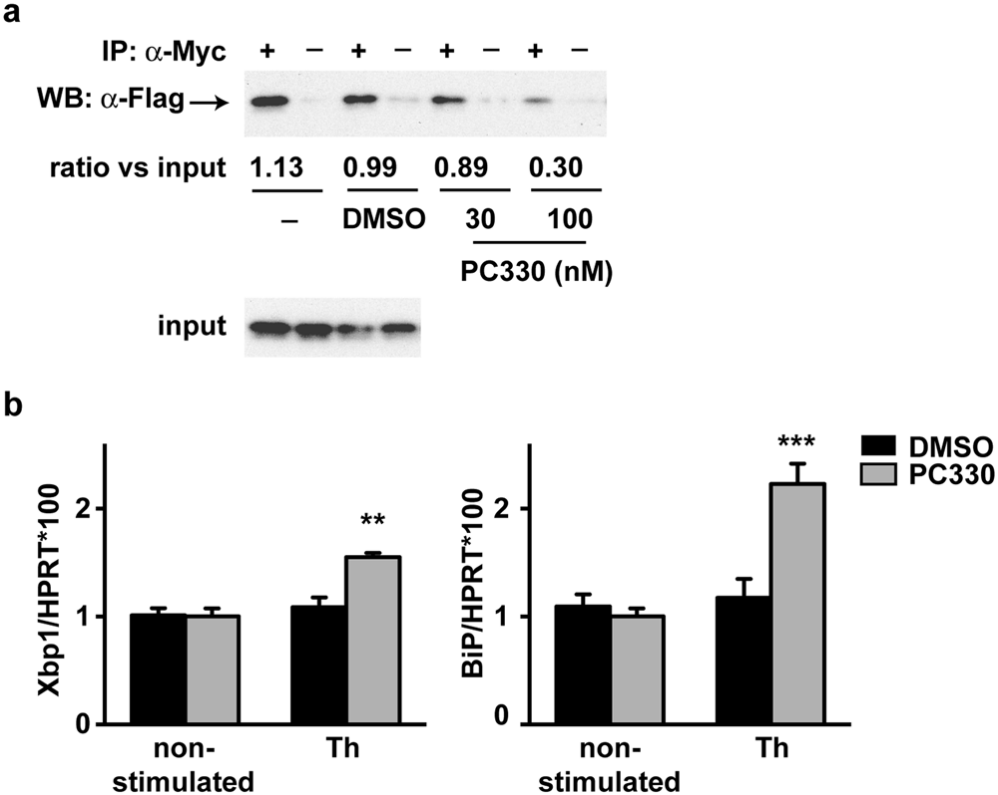
IQM-PC330 promotes ATF6-dependent transcriptional activation. (**a**) Concentration-dependent blockade of DREAM-ATF6 interaction by IQM-PC330. Coimmunoprecipitation of Flag-ATF6 (bZIP fragment aa 306–369) and Myc-DREAM (aa 71–256) proteins after overexpression in HEK293-T cells. Immunoprecipitated band (arrowhead) corresponds to Flag-ATF6. Coimmunoprecipitation was performed in the absence or presence of DMSO or IQM-PC330 (30 and 100 nM). Below, optical density ratio of the ATF6 band relative to the input. (**b)** Real-time qPCR analysis of Xbp1 and BiP mRNAs in STHdhQ^7/7^ cells alone or after stimulation (14 h) with thapsigargin (Th, 50 nM) and in each condition pre-treated with vehicle (DMSO) or IQM-PC330 (PC330, 100 nM; for 1 h). Values are normalized relative to HPRT mRNA levels. The experiment was repeated three times in triplicate. **p = 0.0015 and ***p = 0.0002, (Ordinary Two-way ANOVA, Holm-Sidak multiple comparison test).

Co-immunoprecipitation experiments in the presence of IQM-PC330 showed a concentration-dependent blockade by this compound on the DREAM-ATF6 interaction (Fig. 8a). Then, we checked whether this blockade translates into an increase in ATF6 activity with a higher expression of ATF6 transcriptional target genes. For that, we analyzed the expression levels of ATF6 target genes in thapsigargin-stimulated STHdhQ^7/7^ cells in the absence or the presence of IQM-PC330. As previously shown for repaglinide^11^, exposure to IQM-PC330 enhanced XBP1 and BiP mRNA expression in thapsigargin-stimulated STHdhQ^7/7^ cells exposed to IQM-PC330 (Fig. 8b). These results support the idea of a positive IQM-PC330 action on HD pathology, which prompted us to test the effect after chronic administration to R6/1 mice.

### Chronic administration of IQM-PC330 ameliorates HD symptoms in R6/1 mice

**Figure 9:**
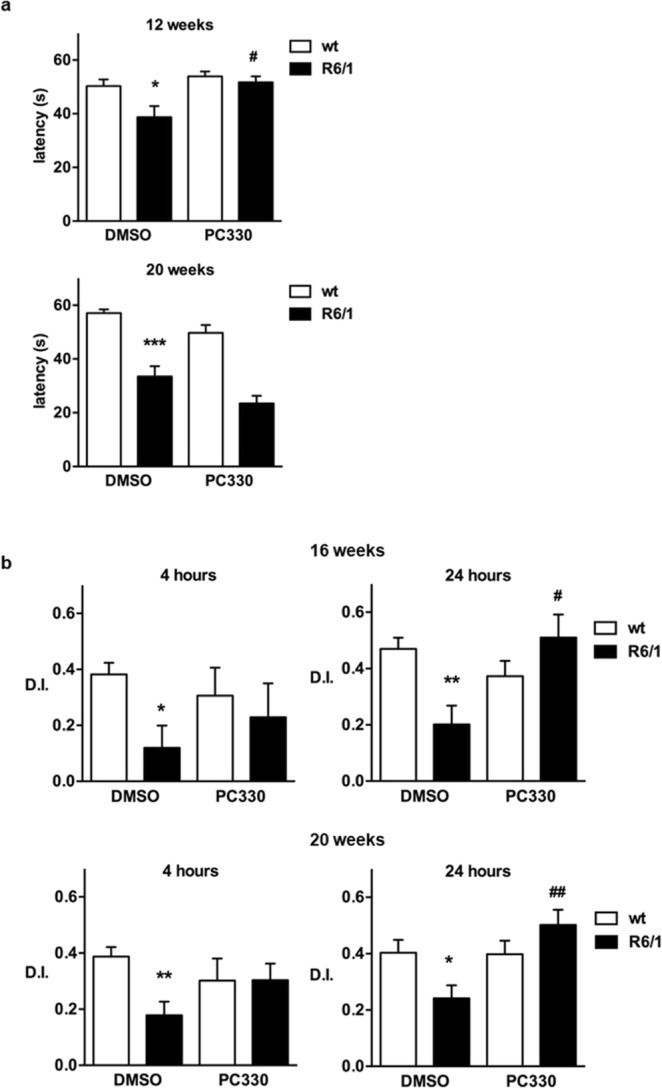
Behavioral assessment of IQM-PC330 effects in R6/1 mice. (**a**) Motor coordination was assessed using the rotarod test. The latency to fall was recorded in 12- and 20-weeks old wt or R6/1 mice. Mice of indicated genotypes received IQM-PC330 or vehicle (DMSO) in drinking water from shortly after weaning. The number of mice used (12 and 20 weeks, respectively): wt-DMSO (20–13), wt-PC330 (21–10), R6/1-DMSO (11–12), R6/1-PC330 (18–12). Data are shown as mean ± SEM. Nonparametric ANOVA, Kruskal-Wallis test (*p* values for both panels <0.0001) with Dunn’s multiple comparisons between selected groups was used. ***p < 0.005, *p < 0.05 vs wt-DMSO; ^#^*p* < 0.05 R6/1-PC330 vs R6/1-DMSO. (**b)** The onset of cognitive impairment was assessed using the novel object recognition test in 16- and 20-weeks old wt or R6/1 mice that received the treatment in the drinking water as in (**a**). For a detailed protocol description see Supporting Information and the Material and Methods section in ref.^11^. The discrimination index (D.I.) reflects the ability to recognize novelty 4 or 24 h after first exposure to the object. The number of mice included in the novel object recognition test (16 and 20 weeks; 4 h and 24 h, respectively): wt-DMSO (27–32, 26–29), wt-PC330 (7–18, 7–16), R6/1-DMSO (21–37, 24–26), R6/1-PC330 (8–18, 9–15) Data are shown as mean ± SEM. Non-parametric ANOVA, Kruskal-Wallis test (*p* values; 16-weeks: 0.05 and 0.005 for 4 and 24 hours, respectively; 20-weeks: 0.003 and 0.009 for 4 and 24 hours, respectively; with Dunn’s multiple comparisons between selected groups was used, **p < 0.01, *p < 0.05 vs wt-DMSO, ^##^*p* < 0.01, ^#^*p* < 0.05 R6/1-PC330 vs R6/1-DMSO.

In R6/1 mice, impaired motor coordination was noticeable by 12 weeks after birth. At this age, R6/1 mice showed a significant reduction in latency to fall in the rotarod test, which was further reduced at 16 and 20 weeks after birth (Fig. 9a). Chronic administration of IQM-PC330 delayed the onset of motor symptoms but had no effect when motor dysfunction was fully established in 20-weeks-old R6/1 mice (Fig. 9a). These results parallel those reported after chronic repaglinide administration^11^ and suggest that, in advanced disease stages, additional mechanisms are recruited and the neuroprotective action of DREAM inhibition on HD-related motor disability is less instrumental.

Accumulating experimental evidence indicates that early changes in cortico-striatal and hippocampal synaptic plasticity are associated with cognitive decline in patients and in mouse models of HD^18–21^. We therefore analyzed the potential effect of IQM-PC330 on learning and memory impairment in R6/1 mice. As reported in several previous studies^12,22,23^, short- and long-term memories were significantly impaired in R6/1 mice when analyzed at 16 and 20 weeks after birth, using the novel object recognition test (Fig. 9b). Chronic administration of IQM-PC330 improved the discrimination index in 16-weeks-old R6/1 mice when tested 4 hours (short term memory) but the effect was only statistically significant when tested 24 hours (long term memory) after the acquisition session (Fig. 9b). Similar results were obtained after chronic administration of IQM-PC330 in 20-weeks-old R6/1 mice (Fig. 9b). In comparable experiments we previously showed that repaglinide did not improve cognition in 20-weeks-old R6/1 mice though it was still effective to reduce the post-prandial increase in circulating glucose levels in these adult R6/1 mice^12^.

These results indicate that IQM-PC330 is active *in vivo* after chronic administration, as it delayed the appearance of HD symptoms in R6/1 mice and showed longer-lasting, more persistent effects than repaglinide. These findings highlight the potential therapeutic applicability of IQM-PC330 or future derivatives for the treatment of neurodegenerative diseases associated with DREAM downregulation.

## Discussion

Reduced DREAM expression or blockade of DREAM activity acts as a neuroprotective mechanism in murine HD models^11,12^. Thus, this protein may be considered a new target in the search of effective HD therapies. Repaglinide, so far the only DREAM ligand tested *in vivo* for HD treatment, has a transient effect; in early disease stages, it effectively delays onset and slows progression of the symptoms, but in the long term does not prevent the fatal outcome of the disease. It is currently not known whether the value of DREAM-associated neuroprotection is masked by additional pathogenic mechanisms at late disease stages, or whether a tolerance mechanism is induced after chronic repaglinide administration. It is nonetheless clear that these results identified an urgent demand for new chemical probes to validate DREAM as therapeutic target in HD and for use as candidates for drug development.

Using a target structure-based design approach, here we identified a series of novel DREAM ligands with improved properties. Thus, IQM-PC330 and IQM-PC332 bound recombinant DREAM *in vitro* (SPR) in a calcium and magnesium concentration-dependent manner and activated DREAM-dependent immediate-early c-fos gene expression. This effect is not caused by an interference of IQM-PC compounds with DREAM oligomerization, at least *in vitro*. Both compounds inhibited the K_V_4.3/DREAM current and blocked DREAM-mediated sensitization of STHdhQ^111/111^ cells. IQM-PC330 was nevertheless 4 times more potent than IQM-PC332 in these *in cellula* experiments. In addition to decrease the K_V_4.3/DREAM current and accelerate its inactivation, both new DREAM ligands delay recovery from inactivation of the K_V_4.3/DREAM current. This effect is not observed with repaglinide nor CL-888 and contributes to the stronger blockade of the *I*_A_ current at high frequencies observed with both IQM-PC compounds.

Computational studies allowed the selection of a cavity centred on Tyr118 and Tyr130, as the ligand-binding pocket. Comparison with the 3D structure of KChIP1-K_V_4.3 complex indicated that these residues are likely to be within the cleft in which the N-terminal helix of K_V_4.3 channel interacts with DREAM. Assessment of the electrophysiological properties of Tyr-to-Ala DREAM mutants supports our predictions of the role of these residues in the channel complex formation and results from cell survival assays confirm their importance for DREAM functional activity.

Endogenous neuroprotection in HD has been associated to an early reduction in DREAM expression. We previously showed that induced DREAM haplodeficiency or inhibition of DREAM activity by repaglinide administration further potentiate the endogenous mechanism(s) of neuroprotection^11,12^. As a result, loss of striatal tissue, impairment of motor coordination and cognitive decline in HD mouse models are delayed. After chronic administration to old R6/1 mice, IQM-PC330 also postponed onset of disease symptoms but had a more persistent effect than repaglinide in blocking long-term cognitive impairment. Several molecular mechanisms might contribute to neuroprotection following IQM-PC330 administration. One relates to an improvement in the pro-survival phase of the UPR by the block of the DREAM-ATF6 interaction and the activation of ATF6 processing. In this regard, here we show that the IQM-PC330 competes with the DREAM-ATF6 interaction in a concentration-dependent manner, at concentrations much lower than those at which repaglinide displaces this protein-protein interaction. Another mechanism, to mention a second signaling pathway in which DREAM also participates, might be associated with the attenuation of the A-type potassium currents by IQM-PC330. It has recently been shown that in R6/2 and zQQ175-KI, two HD mouse models, the striatal neurons forming the indirect pathway show smaller Kir- and voltage-gated K_V_-mediated potassium currents^24^. The reduction in K_V_ channel activity is related to the decrease in DREAM levels and might be part of the endogenous neuroprotective response, though the molecular pathway is presently not known.

Taken together, our results consolidate the pharmacological inhibition of DREAM as a valid therapeutic approach in HD and present a new generation of DREAM inhibitors with improved properties compared to repaglinide. Recent experimental evidence showed an increasing number of molecular commonalities shared by different neurodegenerative diseases^25–27^. Whether the mechanism of DREAM-related neuroprotection in HD is common to other pathologies and whether inhibition of DREAM activity could also be useful in those scenarios remains to be investigated.

## Methods

### Chemistry

For experimental details and description of all new synthetic intermediates, and the characterization of most acetylamino derivatives see the supporting data.

#### Synthesis of IQM-PC330 and IQM-PC332

After 6 h at reflux of the corresponding carboxylic acid (1.5 equiv) in SOCl_2_ (2 mL/mmol), the solution was evaporated to dryness. Then, a solution of the residue in anhydrous THF (2 mL/mmol), the corresponding amine (1.0 equiv) and propylene oxide (5.0 equiv) were stirred overnight at room temperature. After evaporation of the solvent, the crude residue was dissolved in AcOEt (3 × 10 mL), washed with brine (30 mL) and dried over Na_2_SO_4_. The residue was purified as indicated in each case to give the corresponding derivatives bearing an methyl ester (**15** or **32**). Next, a solution of NaOH 2 N (0.22 mL) was added, drop by drop, to a solution of the corresponding ester derivative (1 mmol) in THF/MeOH (1.33 mL/0.66 mL). After 12 hours of stirring at room temperature, the solvent was removed under reduced pressure, water (5 mL) was added and acidified with 1 N HCl at pH 3 or 4. The aqueous phase was extracted with AcOEt (3 × 10 mL). The organic extracts were washed with brine (15 mL), dried over Na_2_SO_4_, evaporated to dryness and lyophilized (H_2_O/CH_3_CN: 1/0.3). The desired final compounds, IQM-PC330 and IQM-PC332, were obtained as an amorphous solid and high purity.

### 2-[2-(3,4-Dichlorophenyl)acetylamino]-4-(4′-n-butylphenyl)benzoic acid (IQM-PC332) (19)

Yield: 68% (two steps). Eluent system: gradient of 0 to 10% of AcOEt in hexane. ^**1**^**H-NMR** (400 MHz, DMSO-*d*_6_) δ (ppm): 0.90 (t, *J* = 7.3 Hz, 3H, CH_3_CH_2_CH_2_CH_2_), 1.31 (sx, *J* = 7.3 Hz, 2H, CH_3_CH_2_CH_2_CH_2_), 1.57 (q, *J* = 7.3 Hz, 2H, CH_3_CH_2_CH_2_CH_2_), 2.62 (t, *J* = 7.3 Hz, 2H, CH_3_CH_2_CH_2_CH_2_), 3.87 (s, 2H, CH_2_CO), 7.31 (d, *J* = 8.3 Hz, 2H, H_3′,5′_), 7.38 (dd, *J* = 8.3, 2.0 Hz, 1H, H_6″_), 7.43 (dd, *J* = 8.3, 1.9 Hz, 1H, H_5_), 7.57 (d, *J* = 8.3 Hz, 2H, H_2′,6′_), 7.62 (d, *J* = 8.3 Hz, 1H, H_5″_), 7.68 (d, *J* = 2.0 Hz, 1H, H_2″_), 8.01 (d, *J* = 8.3 Hz, 1H, H_6_), 8.82 (d, *J* = 1.9 Hz, 1H, H_3_), 11.21 (s, 1H, NH), 13.26–13.78 (bs, OH). ^**13**^**C-NMR** (100 MHz, DMSO-*d*_6_) δ (ppm): 13.8 (CH_3_CH_2_CH_2_CH_2_), 21.8 (CH_3_CH_2_CH_2_CH_2_), 33.0 (CH_3_CH_2_CH_2_CH_2_), 34.5 (CH_3_CH_2_CH_2_CH_2_), 42.9 (CH_2_CO), 115.0 (C_1_), 117.6 (C_3_), 120.8 (C_5_), 126.7 (C_2′,6′_), 129.1 (C_3′,5′_), 129.7 (C_4″_), 130.3 (C_6″_), 130.6 (C_5″_), 130.9 (C_3″_), 131.8 (C_6_), 131.9 (C_2″_), 135.9 (C_1′_), 136.2 (C_1″_), 141.2 (C_2_), 143.0 (C_4′_), 145.4 (C_4_), 168.9 (CO_2_H), 169.3 (CH_2_CO). **HPLC** (Sunfire C18, gradient 80–95% of A in B, 10 min): t_R_ = 4.32 min. **LC-MS (m/z)**: 456.1 ([M + H]^+^). **HRMS (EI**^**+**^**)** m/z found 455.1065 ([M]^+^ C_25_H_23_NO_3_Cl_2_ calculated 455.1055).

### 2-[2-(3,4-Dichlorophenyl)acetylamino]-5-(4’-n-butylphenyl)benzoic acid (IQM-PC330 (36))

Yield: 62% (two steps). Eluent system: gradient of 0 to 10% of AcOEt in hexane. ^**1**^**H-NMR** (400 MHz, DMSO-*d*_6_) δ (ppm): 0.90 (t, *J* = 7.3 Hz, 3H, CH_3_CH_2_CH_2_CH_2_), 1.31 (sx, *J* = 7.3 Hz, 2H, CH_3_CH_2_CH_2_CH_2_), 1.56 (q, *J* = 7.3 Hz, 2H, CH_3_CH_2_CH_2_CH_2_), 2.60 (t, *J* = 7.3 Hz, 2H, CH_3_CH_2_CH_2_CH_2_), 3.85 (s, 2H, CH_2_CO), 7.27 (d, *J* = 8.3 Hz, 2H, H_3′,5′_), 7.38 (dd, *J* = 8.3, 2.0 Hz, 1H, H_6″_), 7.56 (d, *J* = 8.3 Hz, 2H, H_2′,6′_), 7.62 (d, *J* = 8.3 Hz, 1H, H_5′_), 7.68 (d, *J* = 2.0 Hz, 1H, H_2″_), 7.88 (dd, *J* = 8.8, 2.3 Hz, 1H, H_4_), 8.17 (d, *J* = 2.4 Hz, 1H, H_6_), 8.54 (d, *J* = 8.8 Hz, 1H, H_3_), 11.10 (s, 1H, NH). ^**13**^**C-NMR** (100 MHz, DMSO-*d*_6_) δ (ppm): 13.8 (CH_3_CH_2_CH_2_CH_2_), 21.8 (CH_3_CH_2_CH_2_CH_2_), 33.1 (CH_3_CH_2_CH_2_CH_2_), 34.4 (CH_3_CH_2_CH_2_CH_2_), 42.9 (CH_2_CO), 117.3 (C_1_), 120.7 (C_3_), 126.2 (C_2′,6′_), 128.5 (C_6_), 129.0 (C_3′,5′_), 129.7 (C_4″_), 130.2 (C_6″_), 130.6 (C_5″_), 131.0 (C_3″_), 131.8 (C_4_), 131.8 (C_2″_), 131.9 (C_5_), 134.5 (C_1′_), 136.0 (C_1″_), 139.5 (C_2_), 141.8 (C_4′_), 168.7 (CO_2_H), 169.3 (CH_2_CO). **HPLC** (Sunfire C18, gradient 80–95% of A in B, 10 min): t_R_ = 4.58 min. **LC-MS (m/z)**: 456.4 ([M + H]^+^). **HRMS (EI**^**+**^**)** m/z found 455.105 ([M] + C_25_H_23_NO_3_Cl_2_ calculated 455.1055).

### Homology modeling

Homology models of DREAM C-terminal region were built using the NMR structure of the mus musculus DREAM (pdb code 1JUL, 15 structures) and the X-ray structure of the complex between KChIP1-K_V_4.3 (pdb code 2I2R) as the templates. The templates structures were prepared with the Schrödinger Suite of Programs using the Protein Preparation Wizard tool^28,29^, water molecules beyond 5 Å from the protein were deleted. For homology modelling we used the Prime application in Schrödinger Suite 2015^30–32^. Energy minimization was done by using OPLS2005 force field and refinement was carried out until average mean square deviation of the non hydrogen atoms reached 0.3 Å. PROCHECK^33,34^ and Verify3D^35,36^ were used to assess the quality of the models. Using these protocols, six models were generated, five derived from 2JUL (models 1,3,4,7 and 12) and one from 2I2R. These models differ in the three dimensional disposition of some helix, in particular helix 10, which can provide some protein flexibility for the subsequent docking studies.

### Binding site identification

There are not experimentally determined structures that identify a binding pocket for small ligands on the DREAM protein. To ascertain possible binding sites on the homology models generated, we used the SiteMap facility within Schrödinger Suite of Programs^37,38^. A SiteMap calculation identified one or more regions on or near the protein surface, termed sites, which could be suitable for binding of a ligand to DREAM. The top-ranking surface clefts identified by SiteMap were further analyzed by induced fit docking (IFD) studies, using a protocol described below. IFD of the know ligand CL-888 on the six available homology models identified a hydrophobic cleft centered between Tyr118 and Try130, which appeared to be a promising pocket for targeting with small molecules. This binding site was selected for the subsequent IFD studies.

### Ligand preparation

The ligands were constructed using Maestro 2015-4 (Maestro, Schrödinger, LLC, New York, NY, 2015), and ligand structures were prepared using the LigPrep application in the Schrödinger Suite 2015-4^39^. LigPrep optimizes ligand structures, corrects improper bond distances and bond orders and generates ionization states and performs energy minimization.

### Docking studies

The docking site was defined as a cubic box of 10 Å centered on Try118 and Tyr130. Up to 20 poses for ligands were collected. These studies were carried out with the IFD protocol 2015-4^40–42^ within the Schrödinger Suite that allows some protein flexibility. In this protocol, for the initial Glide docking, the van der Waals radii (rdW) of both protein and ligands were scaled to 0.5 to reduce the steric clashes, and the ligands were docked into the fixed DREAM protein. Prime was then used to optimize the side chains of the residues within 5 Å of the ligand poses. Finally, the ligands were re-docked using Glide into the new receptor conformations generated using the default rdW radii. The poses are ranked using IFD score. Selection was based on the best-ranked conformation of each ligand and on visual inspection.

### Molecular dynamic studies

Based on the IFD studies, several ligand-DREAM complexes were selected for further optimization by molecular dynamics simulation, using the Amber 16 and the FF12SB forcefield^43,44^, this allows consideration of the flexibility of the whole complexes. Each ligand-DREAM complex was solvated in an octahedric box using TIP3P water molecules with each site at least 10 Å from any protein atom. The system was neutralized by Na+ counter-ions and periodic boundary conditions were used. Structures were minimized first by steepest descent for 5000 steps, then switched to conjugate gradient for another 5000 steps. The system was subsequently heated gradually from 0 K to 300 K over 100 ps, starting with a positional restraint weight of 5.0 Kcal mol^−1^ A^−2^ and an integration time step of 1.0 fs. In the first 25 ps, the water and counter-ions were equilibrated, while the solutes were restrained. Then, restraints were progressively weaken, and finally removed in the last 100 ps under the NPT ensemble. The temperature was maintained at 300 K using Langevin dynamics. Once the system was equilibrated, 150 ns of production simulation was run, with an integration time step of 2.0 fs and the SHAKE algorithm was used to constrain hydrogen bonds. The particle mesh Ewald method was used to calculate electrostatic interactions. The PyMOL Molecular Graphic System v1.7.0.1 (Schrödinger Inc., München, Germany) was used for visualization and to generate figures^45^.

### Surface plasmon resonance (SPR)

SPR experiments were performed with a Biacore X-100 apparatus (Biacore, GE Healthcare Life Sciences). For a detailed protocol description see Supporting Information and ref.^46^.

#### Binding experiments

The immobilization of the proteins GST-DREAM wild type (WT), and GST-DREAM Y118A and Y130A mutants was carried out on a CM5 sensor chip (Biacore, GE).

#### Affinity experiments

His-DREAM (71–256) was immobilized on a CM4 sensor chip.

### Site-directed mutagenesis

The Tyr-to-Ala DREAM mutants were prepared in the eukaryotic and prokaryotic expression vectors using the QuikChange method (Stratagene).

### Cell culture and transfection

For electrophysiological experiments we used the African green monkey kidney-derived cell line CHO-K1 (CHO), obtained from the American Type Culture Collection (Rockville, MD) and cultured at 37 °C in Iscove’s modified Eagle’s medium supplemented with 10% (v/v) fetal bovine serum (FBS), 1% (v/v) L-Glutamine (Gibco), and antibiotics (100 IU/ml penicillin and 100 μg/ml streptomycin; all from Gibco, Paisley, UK) in a 5% CO_2_ atmosphere^46^. CHO cells were transiently transfected with cDNA encoding K_V_4.3 or K_V_4.3+ DREAM and a reporter plasmid encoding CD8 by using Fugene6 (Promega). Before experimental use, cells were incubated with polystyrene microbeads precoated with anti-CD8 antibody (Thermo Fisher Scientific). In the coimmunoprecipitation experiments, HEK-293-T cells were used, cultured at 37 °C in Dulbecco modified Eagle Medium (DMEM) supplemented as above in a 5% CO_2_ atmosphere.

STHdhQ knock-in striatal neurons^47^ were obtained from Dr. J.J. Lucas (CBM-CSIC, Madrid, Spain). Cells were cultured in DMEM (with 10% FBS, penicillin/streptomycin, Glutamax; all from Invitrogen) and maintained at 32 °C. For experiments, STHdhQ^111/111^ cells were infected with GFP-, DREAM-GFP- or mutant DREAM-expressing lentivirus. Two days after infection, cells were exposed to the mitochondrial toxin hydrogen peroxide (H_2_O_2_, 10 μM) and cell survival was assessed 3 h after toxin exposure using the Cell Proliferation kit I (MTT, Roche). Mycoplasma infection was routinely checked in cell cultures. Whole cell extracts from STHdhQ^7/7^ cells were prepared as described^11^.

### Electrophysiology

Potassium currents were recorded at room temperature (20–25 °C) at a frequency of 0.1 Hz using the whole-cell patch-clamp technique with an Axopatch 200B patch-clamp amplifier (Molecular Devices). For a detailed protocol description see Supporting Information and refs^11,46^. IQM-PC330 and IQM-PC332 were dissolved in DMSO at a stock concentration of 5 × 10^−3^ M and added to the external solution at the desired concentration in each experiment. Origin 8.5 (OriginLab Co.) and Clampfit 10 programs were used to perform least-squares fitting and for data presentation.

### Mice and *in vivo* treatment

R6/1 mice were originally from Jackson Laboratories. For a detailed protocol description see Supporting Information and ref.^11^. IQM-PC330 (2 μg/ml) or vehicle (DMSO, 0.2 μl/ml) was administered chronically in drinking water shortly after weaning.

### Behavioral analysis

Experiments were performed in R6/1 mice and wild-type littermates at indicated ages. For a detailed protocol description see Supporting Information and ref.^11^. Data from the three test trials were averaged for each animal and used in statistical analyses. The Novel Object Recognition test was performed as reported^48,49^.

### Coimmunoprecipitation

HEK293-T cells were cotransfected with plasmids encoding Myc-DREAM_71–256_ and Flag-bZIPATF6_306–369_. For coimmunoprecipitation, whole cell extracts (200 μg) from transfected cells were incubated with 1 μg affinity-purified rabbit anti-Myc (Abcam) in the presence of calcium (2 mM) and vehicle (DMSO) or increasing concentrations of IQM-PC330 (30, 100 and 300 nM). Samples were immunoblotted with mouse anti-Flag (Sigma) and proteins visualized with HRP-conjugated secondary antibody (Jackson) and developed by ECL (ECL Select, GE Healthcare). Blots were quantified using ImageLab software (BioRad).

### Blue native gel electrophoresis

Recombinant His-tagged soluble DREAM protein (aa 71–256) (50 ng, 100 nM) was incubated with increasing concentrations (10, 30, 100 and 300 nM) of IQM-PC330 for 10 min at RT in the presence of CaCl_2_ (5 mM) and directly loaded onto a 10% blue native (nondenaturing, nonreducing) polyacrylamide gel. Samples were mixed with 0.04% Coomassie Brilliant Blue 250G (Merck) before loading and the gel was run with cathode buffer containing 0.001% Coomassie Brilliant Blue 250G. After electrophoresis, gels were incubated in 1% SDS in 50 mM TrisHCl, pH 7.5 before semidry protein transfer onto PVDF membranes (Millipore). Membranes were de-stained in methanol before immunodetection of DREAM proteins using a rabbit polyclonal anti-DREAM antibody^50^.

### Real-time quantitative PCR

Real-time quantitative PCR (qPCR) was performed using RNA isolated from cell as described (See ref.^11^ for more details). Assays from Applied Biosystems were used for the DREAM target gene c-fos (Mm00487425_m1) and for the ATF6 target gene Xbp1 (Mm00457359_m1). For BiP, the primers 5′ ACT TGG AAT GAC CCT TCG GTG 3′ and 5′ TGC TTG TCG CTG GGC ATC 3′ and SYBR technology were used. Target genes were quantified by the normalized expression method using HPRT as reference with primers 5′ TTG GAT ACA GGC CAG ACT TTG TT 3′ and 5′ CTG AAG TAC TCA TTA TAG TCA AGG GCA TA 3′ and the MGB probe 5′ TTG AAA TTC CAG ACA AGT TT 3′, as described (See ref.^11^ for more details).

### Statistical analysis

All data values are shown as mean ± SEM. Differences were considered significant at P < 0.05. When possible, one- or two-way ANOVA was used to analyze statistical differences among groups. In the case of unequal or small sample size or non-Gaussian distribution, comparisons between groups were analyzed using the nonparametric ANOVA, Kruskal-Wallis test with Dunn’s multiple comparisons between groups. Animal experiments were randomized. Sample size was not predetermined by statistical method. Prism GraphPad Software 6.0 was used to plot graphs and for statistical analysis.

### Study approval

Behavioral tests and animal care were conducted in accordance with standard ethical guidelines (European Communities Directive 86/609 EEC; National Institutes of Health 1995) and approved by the National Centre of Biotechnology, Spanish National Research Council and Madrid Community ethical committees (PROEX 028/15).

## Supplementary information

Supplementary Information

## Data Availability

The authors declare that all data supporting the findings of this study are available within the paper and its Supplementary Information files.
