# Supplementary material for: Targeting the neuronal calcium sensor DREAM with small-molecules for Huntington’s disease treatment
Source: Sci Rep. 2019 May 13;9:7260. doi: 10.1038/s41598-019-43677-7 (PMC6514012; doi:10.1038/s41598-019-43677-7)
Supplement: Supplementary file 1 — Supplementary Information [file 41598_2019_43677_MOESM1_ESM.pdf]

SUPPORTING INFORMATION

Targeting the neuronal calcium sensor DREAM with small-molecules for Huntington's disease treatment

Alejandro Lopez-Hurtado[1,2,6] , Diego A. Peraza[3,4,6] , Pilar Cercos[5,6] , Laura Lagartera[5], Paz Gonzalez[1,2], Xose M. Dopazo[1,2], Rosario Herranz[5], Teresa Gonzalez[3,4], Mercedes Martin-Martinez[5], Britt Mellström[1,2], Jose R. Naranjo[1,2] *, Carmen Valenzuela[3,4] *, Marta Gutierrez-Rodriguez[5] *

[1] Spanish Network for Biomedical Research in Neurodegenerative Diseases (CIBERNED), Instituto de Salud Carlos III, Madrid, Spain

[2] Centro Nacional de Biotecnología, CNB-CSIC, Madrid, Spain

[3] Instituto de Investigaciones Biomedicas Alberto Sols, CSIC-UAM. Madrid, Spain.

[4] Spanish Network for Biomedical Research in Cardiovascular Research (CIBERCV), Instituto de Salud Carlos III, Madrid, Spain.

[5] Instituto de Quimica Medica, IQM-CSIC, Madrid, Spain

[6]These authors contributed equally to this work.

* Correspondence and requests for material should be addressed to MGR (e-mail: mgutierrez@iqm.csic.es), CV (e-mail: cvalenzuela@iib.uam.es) and JRN (e-mail: naranjo@cnb.csic.es).

CONTENTS

SI.1 ADDITIONAL EXPERIMENTAL INFORMATION

Supplementary Figure 1……………………………………..S1

Supplementary Figure 2……………………………………...S2

Supplementary Figure 3……………………………………...S3

Supplementary Figure 4……………………………………...S4

Supplementary Table 1 ……………………………………..…S5

SI.2 SYNTHETIC CHEMISTRY PROCEDURES FOR ACETYLAMINO-BENZOIC ACID DERIVATIVES

General Procedures ………………………………………….. S6

A) Synthesis of reference compound IQM-PC205 (2)…... S7

B) Synthesis of acetylamino 4-phenylbenzoic acid derivatives 16-18……………………......………….….…. S7

C) Synthesis of acetylamino 5-phenylbenzoic acid derivatives 33-35………………..……………..………… S14

SI.3 DETAILED PROTOCOL DESCRIPTION

SPR…………………………………………………………….S20

Electrophysiology……………………..……..…………….. S21

Mice and in vivo treatment …………………..………………S21

SI4. REFERENCES……………………………………………………S22

SI.1 ADDITIONAL EXPERIMENTAL INFORMATION

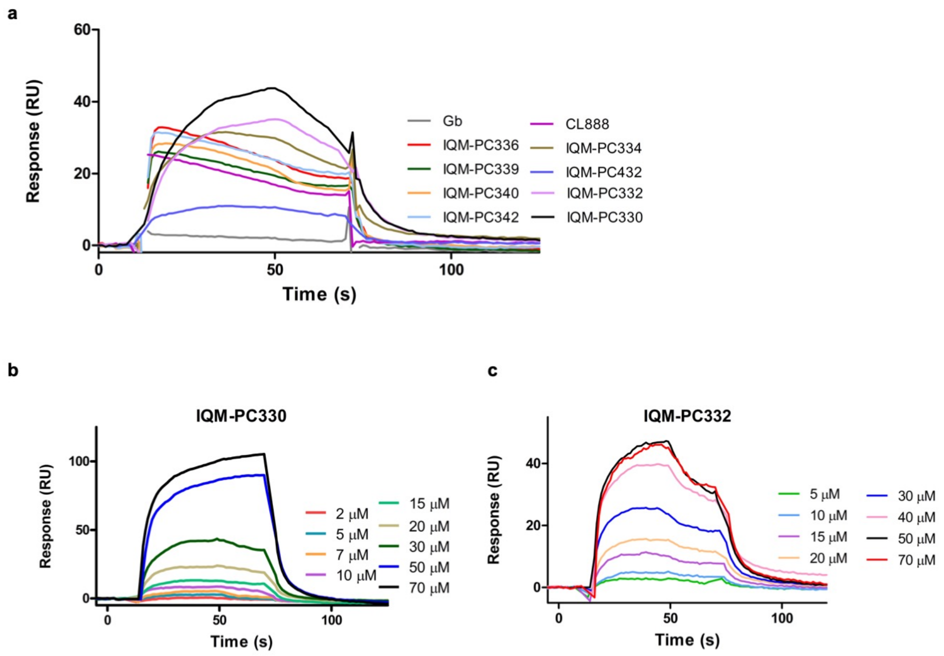

Supplementary Figure 1 (**a**) Sensorgrams for binding of the IQM-compounds to immobilized GST-DREAM. Yes/no binding assays. (**b-c**) Sensorgrams for binding of IQM-PC330 and IQM-PC332 using increasing concentrations of the compounds in the range 5–70 μM to His-DREAM (71-256). RU, resonance units.

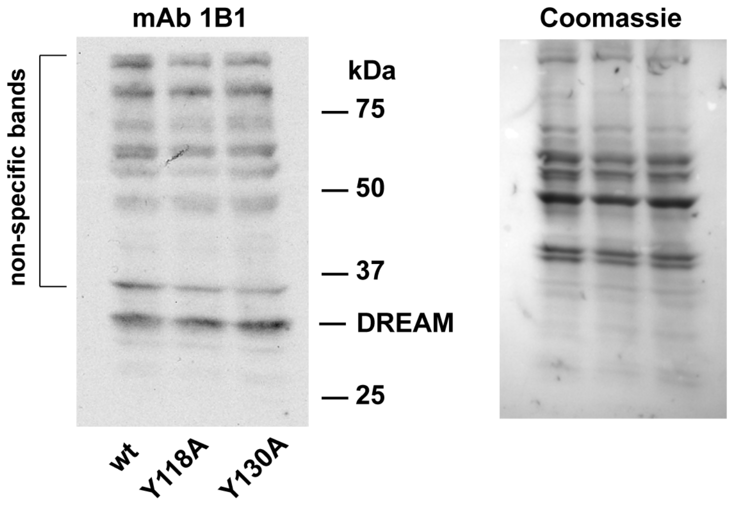

Supplementary Figure 2. Western blot analysis of wild type DREAM, DREAM-Tyr118Ala and DREAM-Tyr130Ala levels following overexpression in STHdhQ111/111 cells (right). Migration of the DREAM protein is indicated. Equal loading of the different lanes was assessed by Coomassie staining (left).

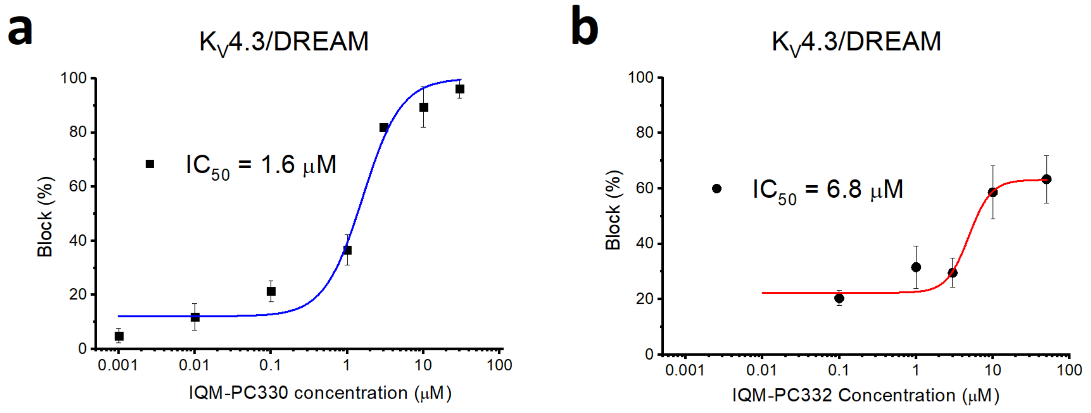

Supplementary Figure 3. (**a-b**) Concentration dependence of IQM-PC330 and IQM-PC332-induced block of K_V4.3/DREAM channels. Block was measured as the inhibition of the amount of charge crossing the membrane.

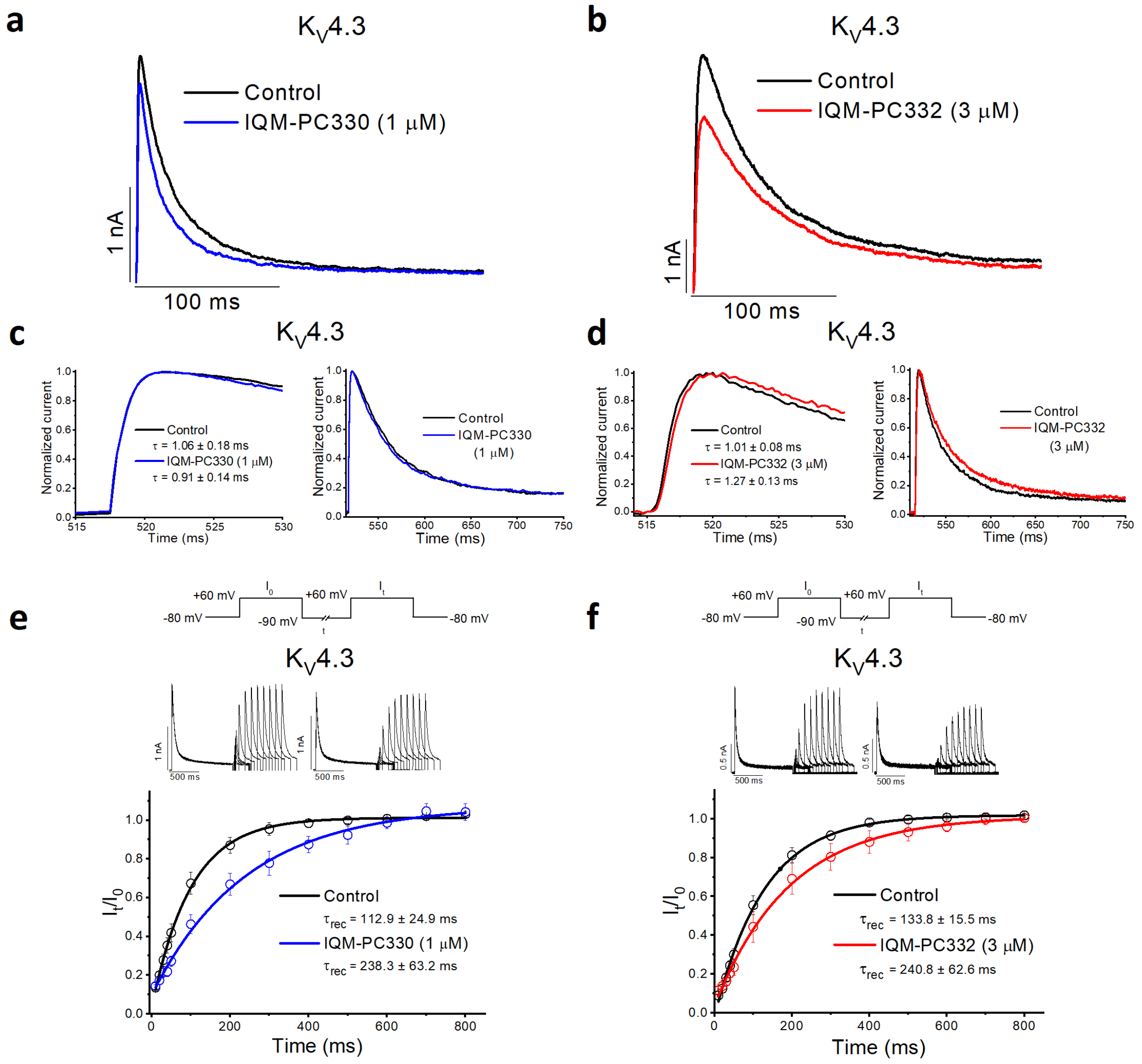

Supplementary Figure 4 **(a-b)** Effects of IQM-PC330 (1 μM) and IQM-PC332 (3 μM) on the activation (left panel) and inactivation (right panel) kinetics of K_V4.3 channels alone. Note that IQM-PC compounds did not modify neither activation nor inactivation **(c-d)** processes. **(e-f)** Effects of IQM-PC330 and IQM-PC332 on the recovery process of K_V4.3 channels. Data are shown as mean ± SEM. A repeated measure ANOVA has been performed followed by a Bonferroni test.

Supplementary Table 1. Kinetic values obtained after fitting the inactivation process to a mono- or biexponential function. Data are expressed as the mean±S.E.M. of n=6-9 experiments. A repeated measure ANOVA has been performed followed by a Bonferroni test. * $p<0.01$.

Experimental condition	Inactivation kinetics	
	τ_f (ms)	τ_s (ms)
**K_V4.3**	29.6 ± 4.8	65.5 ± 6.8
**K_V4.3+PC330 (1 µM)**	21.9 ± 4.1	63.1 ± 6.9
**K_V4.3+DREAM**	53.1 ± 6.4	
**K_V4.3+DREAM+PC330 (1 µM)**	22.7 ± 3.7	104.3 ± 26.4
**K_V4.3**	24.4 ± 1.9	94.5 ± 9.4
**K_V4.3+PC332 (3 µM)**	24.4 ± 1.9	134.5 ± 40.1
**K_V4.3+DREAM**	37.5 ± 4.2	
**K_V4.3+DREAM+PC332 (3 µM)**	44.3 ± 3.2*	
**K_V4.3+DREAM**	34.0 ± 1.4	
**K_V4.3+DREAM+PC332 (100 nM)**	27.1 ± 1.7*	

SI.2 SYNTHETIC CHEMISTRY PROCEDURES FOR ACETYLAMINO-BENZOIC ACID DERIVATIVES

General Procedure 1: Synthesis of amide derivatives

A solution of the corresponding carboxylic acid (1.5 equiv) in $SOCl_2$ (2 mL/mmol) was refluxed, under stirring, for 6h. After this time, excess thionyl chloride was evaporated to dryness. The residue was then dissolved in anhydrous THF (2 mL/mmol), and the corresponding amine (1.0 equiv) and propylene oxide (5.0 equiv) were added to the solution. After stirring overnight at room temperature, the solvent was evaporated to dryness and the crude residue was dissolved in AcOEt (3x10 mL), washed with brine (30 mL) and dried over Na_2SO_4. After removal of the solvent to dryness, the residue was purified by flash chromatography on silica gel, using the eluent mixture indicated in each case.

General Procedure 2: Suzuki-Miyaura coupling reaction

The aryl halide (1.0 equiv), boronic acid (1.5 equiv) and K_2CO_3 (6 equiv) were dissolved in THF/H_2O (5 mL/mmol , 4/1) in a microwave vial. Argon was bubbled for 10 min., then [Pd(PPh_3)$_4$] (2%) was added under a positive pressure of Argon. The vial was sealed, evacuated and backfilled with Argon, and then irradiated in a microwave apparatus at 100 °C, for 30 min. After the reaction mixture was cooled to ambient temperature, the solvent was evaporated to dryness. The resulting solid was resuspended in water (10 mL) and the aqueous phase was extracted with AcOEt (3x10 mL). The organic extracted was dried over Na_2SO_4, filtrated and evaporated to dryness. The crude mixture was purified by flash cromatography using silica gel cartridges and the eluent system indicated in each case.

General Procedure 3: saponification of the ester group.

A solution of NaOH 2N (0.22 mL) was added, drop by drop, to a solution of the corresponding ester derivative (1mmol) in THF/MeOH (1.33 mL/0.66 mL). After 12 hours of stirring at room temperature, the solvent was removed under reduced pressure, water (5mL) was added and acidified with 1N HCl at pH 3 or 4. The aqueous phase was extracted with AcOEt (3 x 10 mL). The organic extracts were washed with brine (15 mL), dried over Na_2SO_4 and evaporated to dryness. The resulting residue was dissolved in H_2O/CH_3CN (3 mL, 1/0.3) and lyophilised. The desired final compounds were obtained as an amorphous solid and high purity.

A) Synthesis of reference compound **IQM-PC205 (2)**

4-Chloro-2-[2-(3,4-dichlorophenyl)acetylamino]benzoic acid

Following General Procedure 1, from 3,4-dichlorophenylacetic acid (504 mg, 3.04 mmol) and 2-amino-4-chlorobenzoic acid (348 mg, 2.03 mmol). After a trituration process with ether as solvent, the title compound was obtained as white amorphous solid (518.2 mg). Yield 73%.

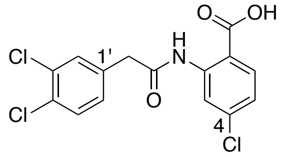

^{1}H-NMR (300 MHz, DMSO-*d*$_6$) δ (ppm): 3.83 (s, 2H, CH_2CO), 7.16 (dd, *J* = 8.6, 2.2 Hz, 1H, H_5), 7.33 (dd, *J* = 8.3, 2.1 Hz, 1H, $H_{6'}$), 7.56 (d, *J* = 8.3 Hz, 1H, $H_{5'}$), 7.62 (d, *J* = 2.1 Hz, 1H, $H_{2'}$), 7.95 (d, *J* = 8.6 Hz, 1H, H_6), 8.61 (d, *J* = 2.2 Hz, 1H, H_3), 11.24 (s, 1H, NH). **^{13}C-NMR** (75 MHz, DMSO-*d*$_6$) δ (ppm): 42.9 ($\underline{C}H_2CO$), 114.8 (C_1), 119.1 (C_3), 122.5 (C_5), 129.8 ($C_{4'}$), 129.9 ($C_{6'}$), 130.4 ($C_{5'}$), 131.0 ($C_{3'}$), 131.6 ($C_{2'}$), 132.6 (C_6), 135.2 ($C_{1'}$), 138.5 (C_4), 141.6 (C_2), 168.6 (CO_2H), 168.9 ($CH_2\underline{C}O$). **HPLC** (Sunfire C18, gradient 50-95% of A in B, 10 min): t_R= 7.61 min. **LC-MS**: 358.5 ($[M+H]^+$). **HRMS (EI$^+$)** *m/z* found 356.9718 ($[M]^+$ $C_{15}H_{10}NO_3Cl_3$ calculated 356.9726).

B) Synthesis of acetylamino 4-phenylbenzoic acid derivatives 16-18.

Methyl 2-amino-4-phenylbenzoate (8)

Following General Procedure 2, from methyl 2-amino-4-bromobenzoate (200 mg, 0.87 mmol) and phenylboronic acid (**4**) (212 mg, 1.74 mmol), the title compound was obtained as white amorphous solid (150 mg). Yield. 76%. Eluent system: gradient of 0 to 10% of AcOEt in hexane.

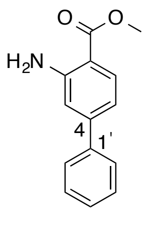

^{1}H-NMR (400 MHz, DMSO-d6) δ (ppm): 3.81 (CO_2CH_3), 6.71 (s, 2H, NH_2), 6.83 (dd, *J* = 8.4, 1.6 Hz, 1H, H_5), 7.06 (td, *J* = 1.6 Hz, 1H, H_3), 7.40 (t, *J* = 7.4 Hz, 1H, $H_{4'}$), 7.47 (t, *J* = 7.4 Hz, 2H, $H_{3',5'}$), 7.61 (d, *J* = 7.4 Hz, 2H, $H_{2',6'}$), 7.78 (d, *J* = 8.4 Hz, 1H, H_6). **^{13}C-NMR** (100 MHz, DMSO-*d*$_6$) δ (ppm): 51.4 ($CO_2\underline{C}H_3$), 107.9 (C_1), 113.6 (C_3),

114.2 (C_5), 126.6 ($C_{3',5'}$), 128.2 ($C_{4'}$), 128.9 ($C_{2',6'}$), 131.3 (C_6), 139.4 (C_4), 145.4 ($C_{1'}$), 151.6 (C_2), 167.6 ($\underline{C}O_2CH_3$). **HPLC** (Sunfire C18, gradient 50-95% of A in B, 10 min): t_R= 3.72 min. **LC-MS**: 228.0 ($[M+H]^+$).

Methyl 2-amino-4-(2'-methylphenyl)benzoate (9)

Following General Procedure 2, from methyl 2-amino-4-bromobenzoate (200 mg, 0.87 mmol) and *o*-tolilboronic acid (**5**) (236 mg, 1.04 mmol), the title compound was obtained as white amorphous solid (170.6 mg). Yield. 81%. Eluent system: gradient of 0 to 10% of AcOEt in hexane.

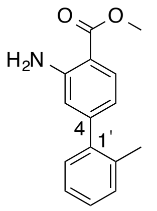

^{1}H-NMR (400 MHz, DMSO-*d*$_6$) δ (ppm): 2.22 (s, 3H, CH_3), 3.80 (s, 3H, CH_3CO_2), 6.49 (dd, *J* = 8.2, 1.7 Hz, 1H, H_5), 6.70 (s, 2H, NH_2), 6.72 (d, *J* = 1.7 Hz, 1H, H_3), 7.14 (dd, *J* = 6.4, 1.8 Hz, 1H, $H_{3'}$), 7.21-7.30 (m, 3H, $H_{4',5',6'}$), 7.73 (d, *J* = 8.2 Hz, 1H, H_7). **^{13}C-NMR** (100 MHz, DMSO-*d*$_6$) δ (ppm): 20.0 (CH_3), 51.4 ($CO_2\underline{C}H_3$), 107,5 (C_1), 116.0 (C_3), 116.7 (C_5), 125.9 ($C_{5'}$), 127.6 ($C_{4'}$), 128.8 ($C_{6'}$), 130.4 ($C_{3'}$), 130.5 (C_6), 134.5 ($C_{2'}$), 140.8 (C_4), 147.0 ($C_{1'}$), 151.1 (C_2), 167.7 ($\underline{C}O_2CH_3$). **HPLC** (Sunfire C18, gradient 50-95% of A in B, 10 min): t_R= 4.75 min. **LC-MS (m/z)**: 241.9 ($[M+H]^+$).

Methyl 2-amino-4-(4'-*tert*-butylphenyl)benzoate (10)

Following General Procedure 2, from methyl 2-amino-4-bromobenzoate (200 mg, 0.87 mmol) and 4-*tert*-butylphenylboronic acid (**6**) (232 mg, 1.31 mmol), the title compound was obtained as white amorphous solid (164.8 mg). Yield: 67%. Eluent system: gradient of 0 to 10% of AcOEt in hexane.

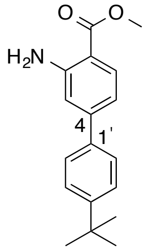

^{1}**H-NMR** (400 MHz, DMSO-*d6*) δ (ppm): 1.31 (s, 9H, $(CH_3)_3C$), 3.80 (s, 3H, CO_2CH_3), 6.72 (s, 2H, NH_2), 6.88 (dd, *J* = 8.4, 1.6 Hz, 1H, H_5), 7.05 (d, *J* = 1.6 Hz, 1H, H_3), 7.48 (d, *J* = 8.1 Hz, 2H, $H_{3',5'}$), 7.55 (d, *J* = 8.1 Hz, 2H, $H_{2',6'}$), 7.76 (d, *J* = 8.4 Hz, 1H, H_6). ^{13}**C-NMR** (100 MHz, DMSO-*d6*) δ (ppm): 31.1 ($(\underline{C}H_3)_3C$), 34.3 ($(CH_3)_3\underline{C}$), 51.4 ($CO_2\underline{C}H_3$), 107.7 (C_1), 113.5 (C_3), 113.9 (C_5), 125.7 ($C_{2',6'}$), 126.3 ($C_{3',5'}$), 131.3 (C_6), 136.5 ($C_{1'}$), 145.3 (C_4), 150.8 ($C_{4'}$), 151.6 (C_2), 167.7 ($\underline{C}O_2CH_3$). **HPLC** (Sunfire C18, gradient 60-95% of A in B, 10 min): t_R= 5.62 min. **LC-MS**: 284.24 ($[M+H]^+$).

Methyl 2-amino-4-(4'-n-butylphenyl)benzoate (11)

Following General Procedure 2, from methyl 2-amino-4-bromobenzoate (360 mg, 1.56 mmol) and 4-n-butylphenylboronic acid (**7**) (418 mg, 2.35 mmol), the title compound was obtained as white amorphous solid (392.5 mg). Yield. 88%. Eluent system: gradient of 0 to 10% of AcOEt in hexane.

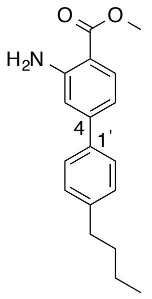

^{1}**H-NMR** (400 MHz, DMSO-*d6*) δ (ppm): 0.90 (t, *J* = 7.3 Hz, 3H, $C\underline{H}_3CH_2CH_2CH_2$), 1.31 (sx, *J* = 7.3 Hz, 2H, $CH_3C\underline{H}_2CH_2CH_2$), 1.56 (q, *J* = 7.3 Hz, 2H, $CH_3CH_2C\underline{H}_2CH_2$), 2.60 (t, *J* = 7.3 Hz, 2H, $CH_3CH_2CH_2C\underline{H}_2$), 3.80 (s, 3H, CH_3CO_2), 6.71 (s, 2H, NH_2), 6.81 (dd, *J* = 8.4, 1.8 Hz, 1H, H_5), 7.05 (d, *J* = 1.8 Hz, 1H, H_3), 7.28 (d, *J* = 8.1 Hz, 2H, $H_{3',5'}$), 7.52 (d, *J* = 8.1 Hz, 2H, $H_{2',6'}$), 7.76 (d, *J* = 8.4 Hz, 1H, H_6). ^{13}**C-NMR** (100 MHz, DMSO-*d6*) δ (ppm): 13.8 ($\underline{C}H_3CH_2CH_2CH_2$), 21.8 ($CH_3\underline{C}H_2CH_2CH_2$), 33.1 ($CH_3CH_2\underline{C}H_2CH_2$), 34.5 ($CH_3CH_2CH_2\underline{C}H_2$), 51.4 ($CO_2\underline{C}H_3$), 107.6 ($C_1$), 113.5 ($C_3$), 113.9 ($C_5$), 126.5 ($C_{2',6'}$), 128.9 ($C_{3',5'}$), 131.3 ($C_6$), 136.7 ($C_{1'}$), 142.5 ($C_4$), 145.4 ($C_{4'}$), 151.6 ($C_2$), 167.7 ($\underline{C}O_2CH_3$). **HPLC** (Sunfire C18, gradient 50-95% of A in B, 10 min): t_R= 6.56 min. **LC-MS**: 284.2 ($[M+H]^+$).

Methyl 2-[2-(3,4-dichlorophenyl)acetylamino]-4-phenylbenzoate (12)

Following General Procedure 1, from **8** (50 mg, 0.22 mmol) and 3,4-dichlorophenylacetic acid (68 mg, 0.33 mmol), the title compound was obtained as white amorphous solid (64.4 mg). Yield. 71%. Eluent system: gradient of 0 to 10% of AcOEt in hexane.

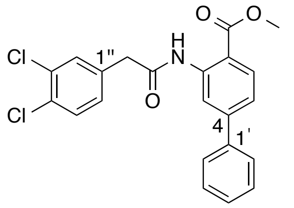

^{1}H-NMR (400 MHz, DMSO-*d_6*) δ (ppm): 3.83 (s, 3H, CO_2CH_3), 3.85 (s, 2H, CH_2CO), 7.37 (dd, *J* = 8.3, 2.0 Hz, 1H, $H_{6''}$), 7.41-7.46 (m, 1H, $H_{4'}$), 7.47-7.53 (m, 3H, $H_{5,3',5'}$), 7.62 (d, *J* = 8.3 Hz, 1H, $H_{5''}$), 7.65-7.69 (m, 3H, $H_{2',6',2''}$), 7.98 (d, *J* = 8.3 Hz, 1H, H_6), 8.56 (d, *J* = 1.8 Hz, 1H, H_3), 10.73 (s, 1H, NH). **^{13}C-NMR** (100 MHz, DMSO-*d_6*) δ (ppm): 42.5 ($\underline{C}H_2CO$), 52.4($CO_2\underline{C}H_3$), 116.8 (C_1), 119.3 (C_3), 121.7 (C_5), 126.9 ($C_{2',6'}$), 128.7 ($C_{4''}$), 129.2 ($C_{3',5'}$), 129.6 ($C_{4'}$), 130.1 ($C_{6''}$), 130.5 ($C_{5''}$), 130.9 ($C_{3''}$), 131.3 (C_6), 131.7 ($C_{2''}$), 136.2 ($C_{1'}$), 138.7 (C_2), 139.9 ($C_{1''}$), 145.3 (C_4), 167.3 ($\underline{C}O_2CH_3$), 168.9 ($CH_2\underline{C}O$). **HPLC** (Sunfire C18, gradient 50-95% of A in B, 10 min): t_R= 8.77 min. **LC-MS**: 414.1 ($[M+H]^+$).

Methyl 2-[2-(3,4-dichlorophenyl)acetylamino]-4-(2’-methylphenyl)benzoate (13)

Following General Procedure 1, from **9** (40 mg, 0.17 mmol) and 3,4-dichlorophenylacetic acid (51 mg, 0.25 mmol), the title compound was obtained as white amorphous solid (33.4 mg). Yield. 47%. Eluent system: gradient of 0 to 20% of AcOEt in hexane.

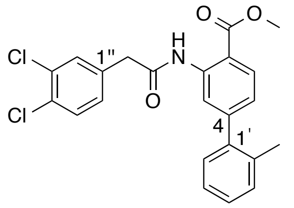

^{1}H-NMR (400 MHz, DMSO-*d_6*) δ (ppm): 2.23 (s, 3H, CH_3), 3.82 (s, 5H, CO_2CH_3, CH_2CO), 7.19 (dd, *J* = 8.1, 1.8 Hz, 1H, H_5), 7.20-7.22 (m, 1H, $H_{6'}$), 7.25-7.30 (m, 1H, $H_{5'}$), 7.30-7.33 (m, 2H, $H_{3',4'}$), 7.35 (dd, *J* = 8.2, 1.9 Hz, 1H, $H_{6''}$), 7.61 (d, *J* = 8.2 Hz, 1H, $H_{5''}$), 7.65 (d, *J* = 1.9 Hz, 1H, $H_{2''}$), 7.95 (d, *J* = 8.1 Hz, 1H, H_6), 8.20 (d, *J* = 1.8 Hz, 1H, H_3), 10.70 (s, 1H, NH). **^{13}C-NMR** (100 MHz, DMSO-*d_6*) δ (ppm): 20.1 (CH_3), 42.5 ($\underline{C}H_2CO$), 52.4 ($CO_2\underline{C}H_3$), 116.6 (C_1), 121.8 (C_3), 126.2 (C_5), 126.2 ($C_{5'}$), 128.1 ($C_{4'}$), 129.2 ($C_{6'}$), 129.6 ($C_{4''}$), 130.1 ($C_{6''}$), 130.5 ($C_{3',5''}$), 130.6 (C_6), 130.9 ($C_{3''}$), 131.7 ($C_{2''}$), 134.6 ($C_{2'}$), 136.2 ($C_{1''}$), 139.1 (C_2), 140.0 ($C_{1'}$), 146.7 (C_4), 167.3 ($\underline{C}O_2CH_3$), 168.8 ($CH_2\underline{C}O$). **HPLC** (Sunfire C18, gradient 50-95% of A in B, 10 min): t_R= 9.23 min. **LC-MS**: 428.3 ($[M+H]^+$).

Methyl 2-[2-(3,4-dichlorophenyl)acetylamino]-4-(4'-*tert*-butylphenyl)benzoate (14)

Following General Procedure 1, from **10** (40 mg, 0.14 mmol) and 3,4-dichlorophenylacetic acid (43 mg, 0.21 mmol), the title compound was obtained as white amorphous solid (45.7 mg). Yield. 69%. Eluent system: gradient of 0 to 20% of AcOEt in hexane.

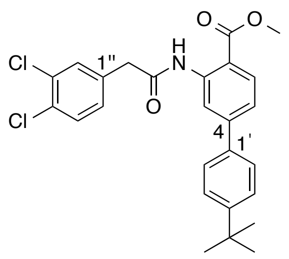

^{1}**H-NMR** (400 MHz, DMSO-d_6) δ (ppm): 1.30 (s, 9H, $(CH_3)_3C$), 3.82 (s, 3H, CO_2CH_3), 3.84 (s, 2H, CH_2CO), 7.37 (dd, *J* = 8.3, 2.0 Hz, 1H, $H_{6''}$), 7.48, (dd, *J* = 8.3, 1.8 Hz, 1H, H_5), 7.51 (d, *J* = 8.6 Hz, 2H, $H_{3',5'}$), 7.60 (d, *J* = 8.6 Hz, 2H, $H_{2',6'}$), 7.62 (d, *J* = 8.3 Hz, 1H, $H_{5''}$), 7.67 (d, *J* = 2.0 Hz, 1H, $H_{2'}$), 7.96 (d, *J* = 8.3 Hz, 1H, H_6), 8.55 (d, *J* = 1,8 Hz, 1H, H_3), 10.74 (s, 1H, NH). ^{13}**C-NMR** (100 MHz, DMSO-d_6) δ (ppm): 31.0 ($(\underline{C}H_3)_3C$), 34.4 ($(CH_3)_3\underline{C}$), 42.5 ($\underline{C}H_2CO$), 52.4 ($CO_2\underline{C}H_3$), 116.5 (C_1), 118.9 (C_3), 121.5 (C_5), 126.0 ($C_{2',6'}$), 126.6 ($C_{3',5'}$), 129.6 ($C_{4''}$), 130.1 ($C_{6''}$), 130.5 ($C_{5''}$), 130.9 ($C_{3''}$), 131.3 (C_6), 131.7 ($C_{2''}$), 135.8 ($C_{1'}$), 136.2 ($C_{1''}$), 139.9 (C_2), 145.2 (C_4), 151.3 ($C_{4'}$), 167.3 ($\underline{C}O_2CH_3$), 168.9 ($CH_2\underline{C}O$). **HPLC** (Sunfire C18, gradient 50-95% of A in B, 10 min): t_R= 6.44 min. **LC-MS (m/z)**: 470.3 ($[M+H]^+$).

Methyl 2-[2-(3,4-dichlorophenyl)acetylamino]-4-(4'-n-butylphenyl)benzoate (15)

Following General Procedure 1, from **11** (60 mg, 0.21 mmol) and 3,4-dichlorophenylacetic acid (65 mg, 0.32 mmol), the title compound was obtained as white amorphous solid (71.5 mg). Yield. 72%. Eluent system: gradient of 0 to 10% of AcOEt in hexane.

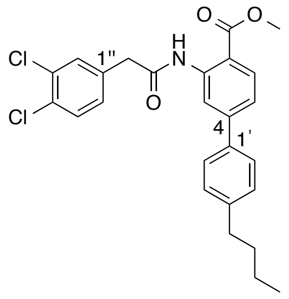

^{1}H-NMR (400 MHz, DMSO-d_6) δ (ppm): 0.90 (t, J = 7.3 Hz, 3H, C$\underline{H}_3$CH$_2$CH$_2$CH$_2$), 1.31 (sx, J = 7.3 Hz, 2H, CH$_3$C$\underline{H}_2$CH$_2$CH$_2$), 1.57 (q, J = 7.3 Hz, 2H, CH$_3$CH$_2$C$\underline{H}_2$CH$_2$), 2.62 (t, J = 7.3 Hz, 2H, CH$_3$CH$_2$CH$_2$C$\underline{H}_2$), 3.83 (s, 3H, CO$_2$CH$_3$), 3.85 (s, 2H, CH$_2$CO), 7.32 (d, J = 8.4 Hz, 2H, H$_{3',5'}$), 7.37 (dd, J = 8.3, 2.1 Hz, 1H, H$_{6''}$), 7.49 (dd, J = 8.3, 1.9 Hz, 1H, H$_5$), 7.58 (d, J = 8.4 Hz, 2H, H$_{2',6'}$), 7.62 (d, J = 8.3 Hz, 1H, H$_{5''}$), 7.67 (d, J = 2.1 Hz, 1H, H$_{2''}$), 7.96 (d, J = 8.3 Hz, 1H, H$_6$), 8.55 (d, J = 1.9 Hz, 1H, H$_3$), 10.73 (s, 1H, NH). **^{13}C-NMR** (100 MHz, DMSO-d_6) δ (ppm): 13.8 ($\underline{C}$H$_3$CH$_2$CH$_2$CH$_2$), 21.8 (CH$_3\underline{C}$H$_2$CH$_2$CH$_2$), 33.0 (CH$_3$CH$_2\underline{C}$H$_2$CH$_2$), 34.5 (CH$_3$CH$_2$CH$_2\underline{C}$H$_2$), 42.5 ($\underline{C}$H$_2$CO), 52.4 (CO$_2\underline{C}$H$_3$), 116.4 (C$_1$), 118.9 (C$_3$), 121.4 (C$_5$), 126.8 (C$_{2',6'}$), 129.1 (C$_{3',5'}$), 129.6 (C$_{4''}$), 130.1 (C$_{6''}$), 130.5 (C$_{5''}$), 130.9 (C$_{3''}$), 131.2 (C$_6$), 131.7 (C$_{2''}$), 136.0 (C$_{1'}$), 136.2 (C$_{1''}$), 139.9 (C$_2$), 143.1 (C$_{4'}$), 145.3 (C$_4$), 167.3 ($\underline{C}$O$_2$CH$_3$), 168.9 (CH$_2\underline{C}$O). **HPLC** (Sunfire C18, gradient 50-95% of A in B, 10 min): t_R= 3.97 min. **LC-MS (m/z)**: 470.2 ([M+H]$^+$).

2-[2-(3,4-Dichlorophenyl)acetylamino]-4-phenylbenzoic acid (16)

Following General Procedure 3, from **12** (29 mg, 0.07 mmol), the title compound was obtained as white amorphous solid (25.5 mg). Yield. 91%.

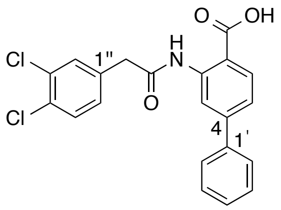

^{1}H-NMR (400 MHz, DMSO-d_6) δ (ppm): 3.87 (s, 2H, CH$_2$CO), 7.38 (dd, J = 8.3, 2.0 Hz, 1H, H$_{6''}$), 7.44 (m, 1H, H$_{4'}$), 7.46 (dd, J = 8.2, 1.8 Hz, 1H, H$_5$), 7.50 (t, J = 7.4 Hz, 2H, H$_{3',5'}$), 7.62 (d, J = 8.3 Hz, 1H, H$_{5''}$), 7.65 (d, J = 2.0 Hz, 1H, H$_{2''}$), 7.68 (dd, J = 7.4, 1.8 Hz, 2H, H$_{2',6'}$), 8.03 (d, J = 8.2 Hz, 1H, H$_6$), 8.83 (d, J = 1.8 Hz, 1H, H$_3$), 11.73 (s, 1H, NH), 13.23-13.89 (bs, OH). **^{13}C-NMR** (100 MHz, DMSO-d_6) δ (ppm): 42.9 ($\underline{C}$H$_2$CO), 115.4 (C$_1$), 117.9 (C$_3$), 121.1 (C$_5$), 126.9 (C$_{2',6'}$), 128.6 (C$_{4''}$), 129.1 (C$_{3',5'}$), 129.7 (C$_{4'}$), 130.3 (C$_{6''}$), 130.5 (C$_{5''}$), 130.9 (C$_{3''}$), 131.8 (C$_6$), 131.9 (C$_{2''}$), 135.9 (C$_{1'}$), 138.9 (C$_2$), 141.1 (C$_{1''}$), 145.4 (C$_4$), 169.0 (CO$_2$H), 169.2 (CH$_2\underline{C}$O). **HPLC** (Sunfire C18, gradient 70-95% de A en B, 10 min): t_R= 2.57 min. **LC-MS**: 400.1 ([M+H]$^+$). **HRMS (EI$^+$)** m/z found 399.0434 ([M]+ $C_{21}H_{15}NO_3Cl_2$ calculated 399.0429).

2-[2-(3,4-Dichlorophenyl)acetylamino]-4-(2'-methylphenyl)benzoic acid (17)

Following General Procedure 3, from **13** (34 mg, 0.08 mmol), the title compound was obtained as white amorphous solid (30.5 mg). Yield. 92%.

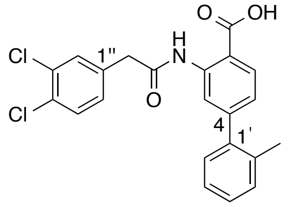

^{1}H-NMR (400 MHz, DMSO-d_6) δ (ppm): 2.23 (s, 3H, CH_3), 3.84 (s, 2H, CH_2CO), 7.13 (dd, J = 8.1, 1.8 Hz, 1H, H_5), 7.16-7.21 (m, 1H, $H_{6'}$), 7.23-7.32 (m, 3H, $H_{5',3',4'}$), 7.36 (dd, J = 8.3, 2.1 Hz, 1H, $H_{6''}$), 7.61 (d, J = 8.3 Hz, 1H, $H_{5''}$), 7.67 (d, J = 2.1 Hz, 1H, $H_{2''}$), 8.01 (d, J = 8.1 Hz, 1H, H_6), 8.49 (d, J = 1.8 Hz, 1H, H_3), 11.20 (s, 1H, NH), 13.34-13.81 (bs, OH). **^{13}C-NMR** (100 MHz, DMSO-d_6) δ (ppm): 20.1 (CH_3), 42.9 ($\underline{C}H_2CO$), 115.1 (C_1), 120.5 (C_3), 123.5 (C_5), 126.1 ($C_{5'}$), 128.0 ($C_{4'}$), 129.1 ($C_{6'}$), 129.7 ($C_{4''}$), 130.3 ($C_{6''}$), 130.5 ($C_{3',5''}$), 130.9 (C_6), 131.0 ($C_{3''}$), 131.9 ($C_{2''}$), 134.6 ($C_{2'}$), 135.9 ($C_{1''}$), 140.2 (C_2), 140.4 ($C_{1'}$), 146.8 (C_4), 168.9 (CO_2H), 169.2 ($CH_2\underline{C}O$). **HPLC** (Sunfire C18, gradient 50-95% of A in B, 10 min): t_R= 6.75 min. **LC-MS (m/z)**: 414.2 ($[M+H]^+$). **HRMS (EI$^+$)** m/z found 413.0603 ($[M]^+$ $C_{22}H_{17}NO_3Cl_2$ calculated 413.0585).

2-[2-(3,4-dichlorophenyl)acetylamino]-4-(4’-*tert*-butylphenyl)benzoic acid (18)

Following General Procedure 3, from **14** (22mg, 0.05 mmol), the title compound was obtained as white amorphous solid (19.8 mg). Yield. 87%.

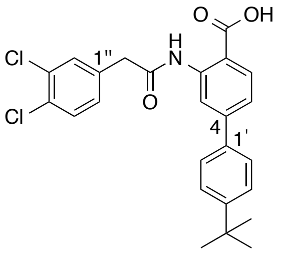

^{1}H-NMR (400 MHz, DMSO-d_6) δ (ppm): 1.31 (s, 9H, $(CH_3)_3C$), 3.87 (s, 2H, CH_2CO), 7.38 (dd, J = 8.3, 2.0 Hz, 1H, $H_{6''}$), 7.44, (dd, J = 8.3, 1.8 Hz, 1H, H_5), 7.52 (d, J = 8.5 Hz, 2H, $H_{3',5'}$), 7.59 (d, J = 8.5 Hz, 2H, $H_{2',6'}$), 7.61 (d, J = 8.3 Hz, 1H, $H_{5''}$), 7.69 (d, J = 2.0 Hz, 1H, $H_{2'}$), 8.02 (d, J = 8.3 Hz, 1H, H_6), 8.55 (d, J = 1,8 Hz, 1H, H_3), 11.24 (s, 1H, NH), 13.43-13.72 (bs, OH). **^{13}C-NMR** (100 MHz, DMSO-d_6) δ (ppm): 31.0 ($(\underline{C}H_3)_3C$), 34.4 ($(CH_3)_3\underline{C}$), 43.0 ($\underline{C}H_2CO$), 115.2 (C_1), 117.6 (C_3), 120.9 (C_5), 125.9 ($C_{2',6'}$), 126.6 ($C_{3',5'}$), 129.7 ($C_{4''}$), 130.3 ($C_{6''}$), 130.6 ($C_{5''}$), 130.9 ($C_{3''}$), 131.8 (C_6), 131.9 ($C_{2''}$), 135.9 ($C_{1'}$), 136.0 ($C_{1''}$), 141.2 (C_2), 145.3 (C_4), 151.2 ($C_{4'}$), 168.9 (CO_2H), 169.2 ($CH_2\underline{C}O$). **HPLC** (Sunfire C18, gradient 50-95% of A in B, 10 min): t_R= 8.06 min. **LC-MS (m/z)**: 456.3 ($[M+H]^+$). **HRMS (EI$^+$)** m/z found 455.1063 ([M]+ $C_{25}H_{23}NO_3Cl_2$ calculated 455.1055).

C) Synthesis of acetylamino 5-phenylbenzoic acid derivatives 33-35,

Methyl 2-amino-5-phenylbenzoate (25)

Following General Procedure 2, from methyl 2-amino-5-bromobenzoate (200 mg, 0.87 mmol) and phenylboronic acid (**21**) (212 mg, 1.74 mmol), the title compound was obtained as white amorphous solid (128.2 mg). Yield. 66%. Eluent system: gradient of 0 to 25% of AcOEt in hexane.

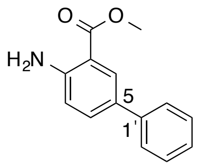

^{1}H-NMR (400 MHz, DMSO-*d6*) δ (ppm): 3.83 (s, 3H, CO_2CH_3), 6.77 (s, 2H, NH_2), 6.89 (d, *J* = 8.7, 1H, H_3), 7.26 (t, *J* = 7.3 Hz, 1H, $H_{4'}$), 7.40 (t, *J* = 7.3 Hz, 2H, $H_{3',5'}$), 7.55 (d, *J* = 7.3 Hz, 2H, $H_{2',6'}$), 7.61 (dd, *J* = 8.7, 2.2 Hz, 1H, H_4), 7.98 (d, *J* = 2.2 Hz, 1H, H_6). **^{13}C-NMR** (100 MHz, DMSO-*d6*) δ (ppm): 51.5 ($CO_2\underline{C}H_3$), 109.0 (C_1), 117.3 (C_3), 125.5 ($C_{2',6'}$), 126.2, 126.7, 128.2, 128.9 ($C_{3',5'}$), 132.5 (C_5), 139.6 ($C_{1'}$), 150.7 (C_2), 167.8 ($\underline{C}O_2CH_3$). **HPLC** (Sunfire C18, gradient 50-95% of A in B, 10 min): t_R= 3.82 min. **LC-MS**: 228.3 ($[M+H]^+$).

Methyl 2-amino-5-(2'-methylphenyl)benzoate (26)

Following General Procedure 2, from methyl 2-amino-5-bromobenzoate (200 mg, 0.87 mmol) and *o*-tolilboronic acid (**22**) (177 mg, 1.31 mmol), the title compound was obtained as colorless oil (79.8 mg). Yield. 76%. Eluent system: gradient of 0 to 10% of AcOEt in hexane.

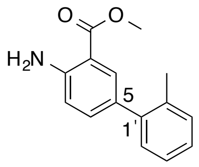

^{1}H-NMR (400 MHz, DMSO-*d6*) δ (ppm): 2.22 (s, 3H, CH_3), 3.78 (s, 3H, CO_2CH_3), 6.72 (s, 2H, NH_2), 6.80 (d, *J* = 8.5 Hz, 1H, H_3), 7.12-7.25 (m, 4H, $H_{3',5'\,6',4'}$), 7.27 (dd, 1H, *J* = 8.5, 2.1 Hz, H_4), 7.63 (d, *J* = 2.1 Hz, 2H, H_6). **^{13}C-NMR** (100 MHz, DMSO-*d6*) δ (ppm): 20.2 (CH_3), 51.4 ($CO_2\underline{C}H_3$), 108.4 (C_1), 116.4 (C_3), 126.7, 127.7 ($C_{4'}$), 129.3 ($C_{6'}$), 130.3 ($C_{3'}$), 130.6 (C_5), 134.7, 134.8 ($C_{2'}$), 141.7 ($C_{1'}$), 150.2 (C_2), 167.7 ($\underline{C}O_2CH_3$). **HPLC** (Sunfire C18, gradient 50-95% of A in B, 10 min): t_R= 4.64 min. **LC-MS (m/z)**: 242.3 ($[M+H]^+$).

Methyl 2-amino-5-(4'-*tert*-butylphenyl)-benzoate (27)

Following General Procedure 2, from methyl 2-amino-5-bromobenzoate (200 mg, 0.87 mmol) and 4-*tert*-butylphenylboronic acid (**23**) (232 mg, 1.31 mmol), the title compound as white amorphous solid (179.6 mg). Yield. 73%. Eluent system: gradient of 0 to 10% of AcOEt in hexane.

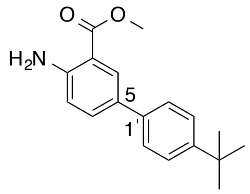

^{1}H-NMR (400 MHz, DMSO-*d6*) δ (ppm): 1.29 (s, 9H, $(CH_3)_3C$), 3.82 (s, 3H, CO_2CH_3), 6.74 (s, 2H, NH_2), 6.87 (d, *J* = 8.6, 1H, H_3), 7.40 (d, *J* = 8.1 Hz, 2H, $H_{3', 5'}$), 7.47 (d, *J* = 8.1 Hz, 2H, $H_{2', 6'}$), 7.58 (dd, *J* = 8.6, 2.4 Hz, 1H, H_4), 7.95 (d, *J* = 2.4 Hz, 1H, H_6). **^{13}C-NMR** (100 MHz, DMSO-*d6*) δ (ppm): 31.2 ($(\underline{C}H_3)_3C$), 34.2 ($(CH_3)_3\underline{C}$), 51.6 ($CO_2\underline{C}H_3$), 109.0 (C_1), 117.4 (C_3), 125.2 ($C_{2',6'}$), 125.7 ($C_{3',5'}$), 126.7, 128.1, 132.5 (C_5), 136.9 ($C_{1'}$), 148.7 ($C_{4'}$), 150.6 (C_2), 167.9 ($\underline{C}O_2CH_3$). **HPLC** (Sunfire C18, gradient 50-95% of A in B, 10 min): t_R= 7.82 min. **LC-MS**: 284.2 ($[M+H]^+$).

Methyl 2-amino-5-(4'-n-butylphenyl)benzoate (28)

Following General Procedure 2, from 2-amino-5-bromobenzoate (360 mg, 1.57 mmol) and 4-n-butylphenylboronic acid (**24**) (418 mg, 2.35 mmol), the title compound was obtained as white amorphous solid (338 mg). Yield. 76%. Eluent system: gradient of 0 to 15% of AcOEt in hexane.

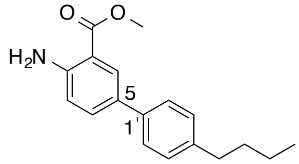

^{1}H-NMR (400 MHz, DMSO-*d6*) δ (ppm): 0.90 (t, *J* = 7.3 Hz, 3H, $C\underline{H}_3CH_2CH_2CH_2$), 1.31 (sx, *J* = 7.3 Hz, 2H, $CH_3C\underline{H_2}CH_2CH_2$), 1.56 (q, *J* = 7.3 Hz, 2H, $CH_3CH_2C\underline{H_2}CH_2$), 2.58 (t, *J* = 7.3 Hz, 2H, $CH_3CH_2CH_2C\underline{H_2}$), 3.82 (s, 3H, CO_2CH_3), 6.75 (s, 2H, NH_2), 6.87 (d, *J* = 8.6, 1H, H_3), 7.21 (d, *J* = 8.1 Hz, 2H, $H_{3',5}$), 7.44 (d, *J* = 8.1 Hz, 2H, $H_{2',6'}$), 7.59 (dd, *J* = 8.6, 2.4 Hz, 1H, H_4), 7.95 (d, *J* = 2.4 Hz, 1H, H_6). **^{13}C-NMR** (100 MHz, DMSO-*d6*) δ (ppm): 13.8 ($\underline{C}H_3CH_2CH_2CH_2$), 21.8 ($CH_3\underline{C}H_2CH_2CH_2$), 33.2 ($CH_3CH_2\underline{C}H_2CH_2$), 34.4 ($CH_3CH_2CH_2\underline{C}H_2$), 51.5 ($CO_2\underline{C}H_3$), 108.9 ($C_1$), 117.3 ($C_3$), 125.3 ($C_{2',6'}$), 126.7, 127.9, 128.8 ($C_{3',5'}$), 132.4 ($C_5$), 137.0 ($C_{1'}$), 140.3 ($C_{4'}$), 150.5 ($C_2$), 167.8 ($\underline{C}O_2CH_3$).

HPLC (Sunfire C18, gradient 50-95% of A in B, 10 min): t_R= 7.81 min. **LC-MS (m/z)**: 284.0 ($[M+H]^+$).

Methyl 2-[2-(3,4-dichlorophenyl)acetylamino]-5-phenylbenzoate (29)

Following General Procedure 1, from **25** (40 mg, 0.18 mmol) and 3,4-dichlorophenylacetic acid (54 mg, 0.26 mmol), the title compound was obtained as white amorphous solid (58.2 mg). Yield. 80%. Eluent system: gradient of 0 to 15% of AcOEt in hexane.

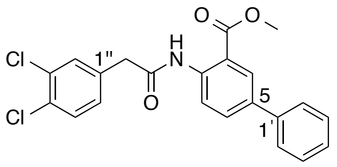

^{1}H-NMR (400 MHz, DMSO-*d$_6$*) δ (ppm): 3.82 (s, 3H, CO_2CH_3), 3.83 (s, 2H, CH_2CO), 7.35-7.40 (m, 2H, $H_{4',6''}$), 7.47 (t, *J* = 7.6 Hz, 2H, $H_{3',5'}$), 7.63 (d, *J* = 8.3 Hz, 1H, $H_{5''}$), 7.65-7.69 (m, 3H, $H_{2',6',2''}$), 7.92 (dd, *J* = 8.7, 2.3 Hz, 1H, H_4), 8.12 (d, *J* = 2.3 Hz, 1H, H_6), 8.25 (d, *J* = 8.7 Hz, 1H, H_3), 10.64 (s, 1H, NH). **^{13}C-NMR** (100 MHz, DMSO-*d$_6$*) δ (ppm): 42.4 ($\underline{C}H_2CO$), 52.4 ($CO_2\underline{C}H_3$), 119.2 (C_1), 122.2 (C_3), 126.4 ($C_{2',6'}$), 127.7 ($C_{4'}$), 128.2 (C_6), 129.1 ($C_{3',5'}$), 129.6 ($C_{4''}$), 130.0 ($C_{6''}$), 130.5 ($C_{2''}$), 130.9 ($C_{3''}$), 131.6 ($C_{5''}$), 131.8 (C_4), 135.2 (C_5), 136.3 ($C_{1''}$), 138.2 ($C_{1'}$), 138.5 (C_2), 167.3 ($\underline{C}O_2CH_3$), 168.7 ($CH_2\underline{C}O$). **HPLC** (Sunfire C18, gradient 50-95% of A in B, 10 min): t_R= 8.50 min. **LC-MS (m/z)**: 414.2 ($[M+H]^+$).

Methyl 2-[2-(3,4-dichlorophenyl)acetylamino]-5-(2'-methylphenyl)benzoate (30)

Following General Procedure 1, from **26** (40 mg, 0.17 mmol) and 3,4-dichlorophenylacetic acid (51 mg, 0.25 mmol), the title compound was obtained as white amorphous solid. Yield. 84%. Eluent system: gradient of 0 to 10% of AcOEt in hexane.

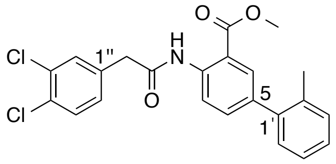

^{1}H-NMR (400 MHz, DMSO-*d$_6$*) δ (ppm): 2.21 (s, 3H, CH_3), 3.78 (s, 3H, CO_2CH_3), 3.83 (s, 2H, CH_2CO), 7.18-7.22 (m, 1H, $H_{6'}$), 7.23-7.32 (m, 3H, $H_{3',4',5'}$), 7.37 (dd, *J* = 8.3, 2.0 Hz, 1H, $H_{6''}$), 7.60 (dd, *J* = 8.5, 2.2 Hz, 1H, H_4), 7.63 (d, *J* = 8.3 Hz, 1H,

H$_{5''}$), 7.67 (d, *J* = 2.0 Hz, 1H, H$_{2''}$), 7.78 (d, *J* = 2.2 Hz, 1H, H$_6$), 8.22 (d, *J* = 8.5 Hz, 1H, H$_3$), 10.64 (s, 1H, NH). **^{13}C-NMR** (100 MHz, DMSO-*d$_6$*) δ (ppm): 20.1 (CH$_3$), 42.4 (C̲H$_2$CO), 52.4 (CO$_2$C̲H$_3$), 118.6 (C$_1$), 121.4 (C$_3$), 126.1 (C$_{5'}$), 127.7 (C$_{4'}$), 129.4 (C$_{6'}$), 129.6 (C$_{4''}$), 130.0 (C$_{6''}$), 130.4 (C$_{3'}$), 130.5 (C$_{5''}$), 130.6 (C$_6$), 130.9 (C$_{3''}$), 131.6 (C$_{2''}$), 134.2 (C$_4$), 134.8 (C$_{2'}$), 136.3 (C$_{5,1''}$), 137.8 (C$_2$), 139.7 (C$_{1'}$), 167.2 (C̲O$_2$CH$_3$), 168.7 (CH$_2$C̲O). **HPLC** (Sunfire C18, gradient 50-95% of A in B, 10 min): t$_R$= 9.28 min. **LC-MS (m/z)**: 428.3 ([M+H]$^+$).

Methyl 2-[2-(3,4-dichlorophenyl)acetylamino]-5-(4'-*tert*-butylphenyl)benzoate (31)

Following General Procedure 1, from **27** (42 mg, 0.15 mmol) and 3,4-dichlorophenylacetic acid (46 mg, 0.22 mmol), the title compound was obtained as white amorphous solid (44.2 mg). Yield. 63%. Eluent system: gradient of 0 to 20% of AcOEt in hexane.

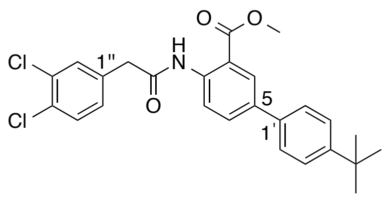

^{1}H-NMR (400 MHz, DMSO-*d$_6$*) δ (ppm): 1.31 (s, 9H, (CH$_3$)$_3$C), 3.82 (s, 3H, CO$_2$CH$_3$), 3.82 (s, 2H, CH$_2$CO), 7.36 (dd, *J* = 8.2, 2.0 Hz, 1H, H$_{6''}$), 7.48 (d, *J* = 8.6 Hz, 2H, H$_{3',5'}$), 7.59 (d, *J* = 8.6 Hz, 2H, H$_{2',6'}$), 7.63 (d, *J* = 8.2 Hz, 1H, H$_{5''}$), 7.66 (d, *J* = 2.0 Hz, 1H, H$_{2''}$), 7.90 (dd, *J* = 8.6, 2.3 Hz, 1H, H$_4$), 8.09 (d, *J* = 2.3 Hz, 1H, H$_6$), 8.22 (d, *J* = 8.6 Hz, 1H, H$_3$), 10.62 (s, 1H, NH). **^{13}C-NMR** (100 MHz, DMSO-*d$_6$*) δ (ppm): 31.1 (C̲H$_3$)$_3$C), 34.3 (CH$_3$)$_3$C̲), 42.4 (C̲H$_2$CO), 52.4 (CO$_2$C̲H$_3$), 119.5 (C$_1$), 122.2 (C$_3$), 125.9 (C$_{2',6'}$), 126.1 (C$_{3',5'}$), 128.0 (C$_6$), 129.6 (C$_{4''}$), 130.0 (C$_{6''}$), 130.5 (C$_{5''}$), 130.9 (C$_{3''}$), 131.6 (C$_4$), 131.6 (C$_{2''}$), 135.2 (C$_5$), 135.7 (C$_{1'}$), 136.3 (C$_{1''}$), 138.0 (C$_2$), 150.2 (C$_{4'}$), 167.3 (C̲O$_2$CH$_3$), 168.7 (CH$_2$C̲O). **HPLC** (Sunfire C18, gradient 80-95% of A in B, 10 min): t$_R$= 6.23 min. **LC-MS (m/z)**: 470.3 ([M+H]$^+$).

Methyl 2-[2-(3,4-dichlorophenyl)acetylamino]-5-(4'-n-butylphenyl)benzoate (32)

Following General Procedure 1, from **28** (40 mg, 0.14 mmol) and 3,4-dichlorophenylacetic acid (43 mg, 0.21 mmol), the title compound was obtained as white amorphous solid (44.5 mg). Yield. 67%. Eluent system: gradient of 0 to 10% of AcOEt in hexane.

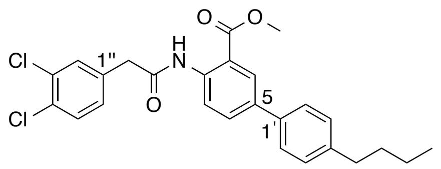

^{1}H-NMR (400 MHz, DMSO-d_6) δ (ppm): 0.90 (t, J = 7.3 Hz, 3H, C$\underline{H}_3$CH$_2$CH$_2$CH$_2$), 1.32 (sx, J = 7.3 Hz, 2H, CH$_3$C$\underline{H}_2$CH$_2$CH$_2$), 1.56 (q, J = 7.3 Hz, 2H, CH$_3$CH$_2$C$\underline{H}_2$CH$_2$), 2.61 (t, J = 7.3 Hz, 2H, CH$_3$CH$_2$CH$_2$C$\underline{H}_2$), 3.82 (s, 3H, CO$_2$CH$_3$), 3.82 (s, 2H, CH$_2$CO), 7.29 (d, J = 8.4 Hz, 2H, $H_{3',5'}$), 7.36 (dd, J = 8.2, 2.0 Hz, 1H, $H_{6''}$), 7.57 (d, J = 8.4 Hz, 2H, $H_{2',6'}$), 7.64 (d, J = 8.2 Hz, 1H, $H_{5''}$), 7.66 (d, J = 2.0 Hz, 1H, $H_{2''}$), 7.89 (dd, J = 8.7, 2.3 Hz, 1H, H_4), 8.09 (d, J = 2.3 Hz, 1H, H_6), 8.23 (d, J = 8.7 Hz, 1H, H_3), 10.62 (s, 1H, NH). **^{13}C-NMR** (100 MHz, DMSO-d_6) δ (ppm): 13.8 ($\underline{C}$H$_3$CH$_2$CH$_2$CH$_2$), 21.7 (CH$_3$$\underline{C}H_2CH_2CH_2$), 33.1 (CH$_3CH_2$$\underline{C}H_2CH_2$), 34.1 (CH$_3CH_2CH_2$$\underline{C}H_2$), 42.4 ($\underline{C}H_2$CO), 52.4 (CO$_2$$\underline{C}H_3$), 119.2 ($C_1$), 122.2 ($C_3$), 126.2 ($C_{2',6'}$), 127.9 ($C_6$), 129.0 ($C_{3',5'}$), 129.6 ($C_{4''}$), 130.0 ($C_{6''}$), 130.5 ($C_{5''}$), 130.9 ($C_{3''}$), 131.5 ($C_4$), 131.6 ($C_{2''}$), 135.2 ($C_5$), 135.8 ($C_{1'}$), 136.3 ($C_{1''}$), 138.0 ($C_2$), 142.0 ($C_{4'}$), 167.3 ($\underline{C}O_2CH_3$), 168.6 (CH$_2$$\underline{C}$O). **HPLC** (Sunfire C18, gradient 80-95% of A in B, 10 min): t_R= 7.15 min. **LC-MS (m/z)**: 470.2 ([M+H]$^+$).

2-[2-(3,4-Dichlorophenyl)acetylamino]-5-phenylbenzoic acid (33)

Following General Procedure 3, from **29** (35 mg, 0.08 mmol), the title compound was obtained as white amorphous solid (33.5 mg). Yield. 98%.

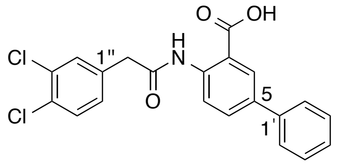

^{1}H-NMR (400 MHz, DMSO-d_6) δ (ppm): 3.86 (s, 2H, CH$_2$CO), 7.34-7.42 (m, 2H, $H_{4',6''}$), 7.47 (t, J = 7.6 Hz, 2H, $H_{3',5'}$), 7.62 (d, J = 8.2 Hz, 1H, $H_{5''}$), 7.64-7.71 (m, 3H, $H_{2',6',2''}$), 7.91 (dd, J = 8.7, 2.4 Hz, 1H, H_4), 8.20 (d, J = 2.4 Hz, 1H, H_6), 8.56 (d, J = 8.7 Hz, 1H, H_3), 11.14 (s, 1H, NH), 13.56-13.93 (bs, OH). **^{13}C-NMR** (100 MHz, DMSO-d_6) δ (ppm): 42.9 ($\underline{C}$H$_2$CO), 117.4 (C_1), 120.7 (C_3), 126.3 ($C_{2',6'}$), 127.6 ($C_{4'}$), 128.7 (C_6), 129.1 ($C_{3',5'}$), 129.7 ($C_{4''}$), 130.2 ($C_{6''}$), 130.6 ($C_{2''}$), 130.9 ($C_{3''}$), 131.8 ($C_{5''}$), 132.0 (C_4), 134.5 (C_5), 136.0 ($C_{1''}$), 138.6 ($C_{1'}$), 139.8 (C_2), 168.7 (CO$_2$H), 169.2 (CH$_2$$\underline{C}$O). **HPLC** (Sunfire C18, gradient 50-95% of A in B, 10 min): t_R= 5.95 min. **LC-MS (m/z)**: 400.1 ([M+H]$^+$). **HRMS (EI$^+$)** m/z found 399.0417 ([M]+ $C_{21}H_{15}NO_3Cl_2$ calculated 399.0429).

2-[2-(3,4-Dichlorophenyl)acetylamino]-5-(2'-methylphenyl)benzoic acid (34)

Following General Procedure 3, from **30** (35 mg, 0.08 mmol), the title compound was obtained as white amorphous solid (31 mg). Yield. 91%.

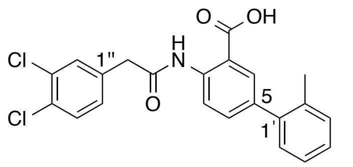

^{1}H-NMR (400 MHz, DMSO-*d$_6$*) δ (ppm): 2.22 (s, 3H, CH_3), 3.86 (s, 2H, CH_2CO), 7.20 (m, 1H, $H_{6'}$), 7.22-7.32 (m, 3H, $H_{3',4',5'}$), 7.38 (dd, *J* = 8.3, 2.1 Hz, 1H, $H_{6''}$), 7.59 (dd, *J* = 8.6, 2.2 Hz, 1H, H_4), 7.62 (d, *J* = 8.3 Hz, 1H, $H_{5''}$), 7.69 (d, *J* = 2.1 Hz, 1H, $H_{2''}$), 7.86 (d, *J* = 2.2 Hz, 1H, H_6), 8.53 (d, *J* = 8.6 Hz, 1H, H_3), 11.12 (s, 1H, NH), 13.40-13.79 (bs, OH). **^{13}C-NMR** (100 MHz, DMSO-*d$_6$*) δ (ppm): 20.1 (CH_3), 42.9 ($\underline{C}H_2CO$), 116.7 (C_1), 120.0 (C_3), 126.1 ($C_{5'}$), 127.6 ($C_{4'}$), 129.4 ($C_{6'}$), 129.7 ($C_{4''}$), 130.2 ($C_{6''}$), 130.5 ($C_{3'}$), 130.6 ($C_{5''}$), 131.0 (C_6), 131.2 ($C_{3''}$), 131.8 ($C_{2''}$), 134.5 (C_4), 134.7 ($C_{2'}$), 135.6 (C_5), 136.0 ($C_{1''}$), 139.3 (C_2), 139.8 ($C_{1'}$), 168.7 (CO_2H), 169.2 ($CH_2\underline{C}O$). **HPLC** (Sunfire C18, gradient 50-95% of A in B, 10 min): t_R= 6.66 min. **LC-MS (m/z)**: 414.2 ($[M+H]^+$). **HRMS (EI$^+$)** m/z found 413.05998 ([M]+ $C_{22}H_{17}NO_3Cl_2$ calculated 413.05855).

2-[2-(3,4-Dichlorophenyl)acetylamino]-5-(4'-*tert*-butylphenyl)benzoic acid (35)

Following General Procedure 3, from **31** (20 mg, 0.04 mmol), the title compound was obtained as white amorphous solid (17.5 mg). Yield. 96%.

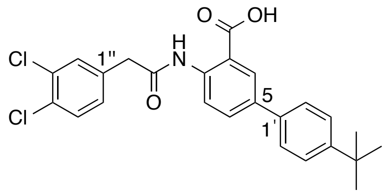

^{1}H-NMR (400 MHz, DMSO-*d$_6$*) δ (ppm): 1.30 (s, 9H, $(CH_3)_3C$), 3.88 (s, 2H, CH_2CO), 7.38 (dd, *J* = 8.3, 2.1 Hz, 1H, $H_{6''}$), 7.47 (d, *J* = 8.6 Hz, 2H, $H_{3',5'}$), 7.58 (d, *J* = 8.6 Hz, 2H, $H_{2',6'}$), 7.62 (d, *J* = 8.3 Hz, 1H, $H_{5''}$), 7.68 (d, *J* = 2.1 Hz, 1H, $H_{2''}$), 7.88 (dd, *J* = 8.6, 2.4 Hz, 1H, H_4), 8.18 (d, *J* = 2.4 Hz, 1H, H_6), 8.53 (d, *J* = 8.6 Hz, 1H, H_3), 11.17 (s, 1H, NH), 13.46-13.95 (bs, OH). **^{13}C-NMR** (100 MHz, DMSO-*d$_6$*) δ (ppm): 31.0 ($(\underline{C}H_3)_3C$), 34.3 ($(CH_3)_3\underline{C}$), 42.9 ($\underline{C}H_2CO$), 118.2 (C_1), 121.3 (C_3), 126.5 ($C_{2',6'}$), 126.7 ($C_{3',5'}$), 129.2 (C_6), 130.3 ($C_{4''}$), 130.9 ($C_{6''}$), 131.3 ($C_{5''}$), 131.6 ($C_{3''}$), 132.4 (C_4), 132.5 ($C_{2''}$), 135.0 (C_5), 136.5 ($C_{1'}$), 136.7 ($C_{1''}$), 140.2 (C_2), 150.0 ($C_{4'}$), 169.4 (CO_2H), 169.9 ($CH_2\underline{C}O$). **HPLC** (Sunfire C18, gradient 50-95% of A in B, 10 min): t_R= 8.77 min. **LC-MS (m/z)**: 456.1 ($[M+H]^+$). **HRMS (EI$^+$)** m/z found 455.1049 ([M]+ $C_{25}H_{23}NO_3Cl_2$ calculated 455.1055).

SI3. DETAILED PROTOCOL DESCRIPTION

Surface plasmon resonance (SPR)[1]

Binding experiments. SPR experiments were performed at room temperature (20 ºC) with a Biacore X-100 apparatus (Biacore, GE Healthcare Life Sciences) in running buffer (50 mM Tris pH 7.5, 50 mM NaCl, 2 mM CaCl2 with 2% dimethylsulfoxide and 0.05% Tween 20). For calcium experiments the buffer used was the same without calcium. The proteins GST-DREAM wild type (WT), and GST-DREAM Y118A and Y130A mutants were immobilized on a CM5 sensor chip (Biacore, GE) following a standard amine coupling method[2]. A 7-min injection of a 1:1 ratio of 0.4M EDC (1-ethyl-3-(3-dimethylaminopropyl)carbodiimide hydrochloride) and 0.1M *N*-hydroxysuccinimide was used to activate the carboxymethyl dextran surface of the experimental flow cell. The corresponding protein was coupled to the surface with a 7-min injection at several dilutions at 10-100μg/ml in 10mM sodium acetate, pH 4.0. Unreacted *N*-hydroxysuccinimide esters were quenched by a 7-min injection of 0.1 M ethanolamine-HCl (pH 8.0). Inmobilization levels were in the 6.000-8.000 RUs range. Reference flow cell was treated as experimental flow cell (amine coupling procedure) but without protein. Prior to use, 10mM stock solutions of both compounds were diluted several times to a 5-10 μM final concentration in running buffer. Typically, a series of different compounds was injected onto the sensor chip at a 90 μl/min flow rate for 1min, followed by a 1 min dissociation period. After dissociation, an extra wash was done over the flow cells with 50% DMSO. No regeneration was needed.

Affinity experiments. His-DREAM (71-256) was immobilized on a CM4 sensor chip as the conditions described previously for GST-DREAM. The immobilization level was 6.000 RUs. For affinity experiments IQM-PC330 and IQM-PC332 were diluted several times to a 5-70μM final concentration in running buffer (supplied until 5 mM of $CaCl_2$ for IQM-PC330 experiments). The measurements were made at the same conditions of binding experiments using 5-70μM range concentrations.

Data processing. Sensograms data were double-referenced and solvent-corrected using the Biaevaluation X-100 software (Biacore, GE Healthcare Life Sciences). Experimental data for affinity measurements were adjusted to a one site-specific model binding with Hill slope using the equation: $R_{eq} = R_{max}[A]^n/(K_D^n + [A]^n)$; where R_{eq} is the equilibrium response at each concentration, R_{max} is the maximum specific binding, [A] is the analyte concentration, K_D the equilibrium dissociation constant and n the hill slope.

Electrophysiology[1,3]

Potassium currents were recorded at room temperature (20-25ºC) at a frequency of 0.1 Hz using the whole-cell patch-clamp technique with an Axopatch 200B patch-clamp amplifier (Molecular Devices). Currents were filtered at 2 kHz (four-pole Bessel filter) and sampled at 4 kHz. Micropipettes were pulled from borosilicate glass capillary tubes (Narishige GD-1) on a programmable horizontal puller (Sutter Instrument Co.) and heat-polished with a microforge (Narishige, Japan). Micropipette resistance was 1.8-3 MΩ. Capacitance and series resistance compensation were optimized, with 80% compensation of the effective access resistance usually obtained. The intracellular pipette filling solution contained: 80 mM K-aspartate, 42 mM KCl, 3 mM phosphocreatine, 10 mM KH_2PO_4, 3 mM MgATP, 5 mM HEPES-K, 5 mM EGTA-K and it was adjusted to pH 7.25 with KOH. The bath solution contained: 136 mM NaCl, 4 mM KCl, 1.8 mM $CaCl_2$, 1 mM $MgCl_2$, 10 mM HEPES-Na and 10 mM glucose and it was adjusted to pH 7.40 with NaOH. IQM-PC330 and IQM-PC332 were dissolved in DMSO at a stock concentration of 5×10^{-3} M and added to the external solution at the desired concentration in each experiment. Origin 8.5 (OriginLab Co.) and Clampfit 10 programs were used to perform least-squares fitting and for data presentation.

Mice and *in vivo* treatment[3,4]

R6/1 mice were originally from Jackson Laboratories. Our colonies were maintained by breeding R6 mice with CBA × C57BL/6 mice to obtain heterozygous mutants and wild-type offspring. Genotype and CAG-repeat length were determined by PCR-based amplification using genomic DNA extracted from tail biopsies. IQM-PC330 (2 μg/ml) or vehicle (DMSO, 0.2 μl/ml) was administered chronically in drinking water shortly after weaning.

Behavioral analysis[3-6]

Experiments were performed in R6/1 mice and wild-type littermates. Motor coordination was assessed in 12- and 20-weeks wt or R6/1 old mice using the rotarod test. Mice of indicated genotypes and ages were exposed chronically to vehicle (DMSO) or IQM-PC330. The number of mice used (12 and 20 weeks, respectively): wt-DMSO (20-13), wt-PC330 (21-10), R6/1-DMSO (11-12), R6/1-PC330 (18-12). Memory was assessed in 16- and 20-weeks wt or R6/1 old mice using the novel object recognition test. Mice received IQM-PC330 or vehicle (DMSO) in drinking water from shortly after weaning. The discrimination index (D.I.) reflects the ability to recognize novelty 4 or 24 h after first exposure to the object. The number of mice included in the novel object recognition test (16 and 20 weeks; 4 h and 24 h, respectively): wt-DMSO

(27-32, 26-29), wt-PC330 (7-18, 7-16), R6/1-DMSO (21-37, 24-26), R6/1-PC330 (8-18, 9-15).

Mice were initially housed five per cage in a temperature- (21 ± 1°C) and humidity- (65 ± 10%) controlled room with a 12/12-h light/dark cycle (lights on from 0800 to 2000 h), with food and water *ad libitum*. All experiments took place during the light phase. All behavioral experiments were carried out in blind conditions for genotype and treatment.

The rotarod test was used to measure motor coordination and balance (Accelerating Model, Ugo Basile, Biological Research Apparatus). For basal rotarod performance, mice were tested on two consecutive days. On day 1 (training), each mouse was placed on the rotarod at a constant speed (4 r.p.m.) for a maximum of 60 s. The procedure was repeated three times with a rest period of 30 min between trials. On day 2 (experiment), mice received one training trial at constant speed (4 r.p.m.) for a maximum of 60 s, followed by three test trials with acceleration from 4 to 40 r.p.m. over a period of 60 s, and the latency to fall off the rotarod within this time period was recorded. Any mice remaining on the apparatus after 60 s were removed and their time scored as 60 s. Data from the three test trials were averaged for each animal and used in statistical analyses.

The Novel Object Recognition test was performed as reported[5,6]. In brief, mice were first individually habituated to the open-field for 50 min. The next day, they were submitted to a 10-min acquisition trial (first trial) during which they were placed in the open-field in the presence of object A. The time that mice took to explore object A (animal's snout directed toward the object at a distance < 1 cm) was recorded. Two 10-min retention trials occurred 4 h later (second trial) and 24 h later (third trial). During second and third trials, objects A and B (second trial) or A and C (third trial) were placed in the open-field, and the time that animal took to explore object A (**t**A) and the novel objects B or C, respectively, (**t**N) were recorded. A discrimination index was defined as [**t**N - **t**A/(**t**A + **t**N)]. Mice that explored for less than 5 s during the initial 10-min acquisition trial were excluded from the test. Similarly, mice that explored for < 5 s during a given retention trial (4 or 24 h) were excluded from that analysis.

SI4. REFERENCES

1. Peraza, D. A. et al. Identification of IQM-266, a novel DREAM ligand that modulates K_V4 currents. *Front. Mol. Neurosci.* **12,** 1-11 (2019).

2. Johnsson, B., Loefaas, S. & Lindquist, G. Immobilization of proteins to a carboxymethyldextran-modified gold surface for biospecific interaction analysis in surface plasmon resonance sensors. *Anal. Biochem.* **198**, 268-277 (1991).

3. Naranjo, J.R. et al. Activating transcription factor 6 derepression mediates neuroprotection in Huntington disease. *J. Clin. Invest.* **126**, 627-638 (2016).

4. Lopez-Hurtado, A. et al. Inhibition of DREAM-ATF6 interaction delays onset of cognition deficit in a mouse model of Huntington's disease. *Mol. Brain* **11**, 13 (2018).

5. Tan, V.T.Y. et al. Lentivirus-mediated expression of human secreted amyloid precursor protein-alpha prevents development of memory and plasticity deficits in a mouse model of Alzheimer's disease. *Mol. Brain* **11**, 7 (2018).

6. Cui, L. et al. Disrupted-in-schizophrenia1 (DISC1) L100P mutation alters synaptic transmission and plasticity in the hippocampus and causes recognition memory deficits. *Mol. Brain* **9**, 89-101 (2016).
